# Identifying and modeling the structural discontinuities of human interactions

**DOI:** 10.1038/srep46677

**Published:** 2017-04-26

**Authors:** Sebastian Grauwin, Michael Szell, Stanislav Sobolevsky, Philipp Hövel, Filippo Simini, Maarten Vanhoof, Zbigniew Smoreda, Albert-László Barabási, Carlo Ratti

**Affiliations:** 1 Senseable City Lab, Massachusetts Institute of Technology, 77 Massachusetts Avenue, Cambridge, MA 02139, USA; 2 Center for Complex Network Research, Northeastern University, 110 Forsyth Street, Boston, MA 02115, USA; 3 Hungarian Academy of Sciences, Centre for Social Sciences, Tóth Kálmán utca 4, 1097 Budapest, Hungary; 4 Center for Urban Science And Progress, New York University, 1 MetroTech Center, 19fl, Brooklyn, NY 11201, USA; 5 Institut für Theoretische Physik, Technische Universität Berlin, Hardenbergstraße 36, 10623 Berlin, Germany; 6 Bernstein Center for Computational Neuroscience, Humboldt-Universität zu Berlin, Philippstraße 13, 10115 Berlin, Germany; 7 Department of Engineering Mathematics, University of Bristol, Woodland Road, Bristol, BS8 1UB, United Kingdom; 8 Institute of Physics, Budapest University of Technology and Economics, Budafokiút 8, Budapest, H-1111, Hungary; 9 Département SENSE, Orange Labs, 38 rue du Général Leclerc, 92794 Issy-les-Moulineaux, France; 10 Center for Cancer Systems Biology, Dana-Farber Cancer Institute, Boston, Massachusetts 02115, USA; 11 Department of Medicine, Brigham and Women’s Hospital, Harvard Medical School, Boston, Massachusetts 02115, USA

## Abstract

The idea of a hierarchical spatial organization of society lies at the core of seminal theories in human geography that have strongly influenced our understanding of social organization. Along the same line, the recent availability of large-scale human mobility and communication data has offered novel quantitative insights hinting at a strong geographical confinement of human interactions within neighboring regions, extending to local levels within countries. However, models of human interaction largely ignore this effect. Here, we analyze several country-wide networks of telephone calls - both, mobile and landline - and in either case uncover a systematic decrease of communication induced by borders which we identify as the missing variable in state-of-the-art models. Using this empirical evidence, we propose an alternative modeling framework that naturally stylizes the damping effect of borders. We show that this new notion substantially improves the predictive power of widely used interaction models. This increases our ability to understand, model and predict social activities and to plan the development of infrastructures across multiple scales.

Globalization has led us to believe that our world is becoming borderless and deterritorialized. The rise of novel information technologies has even prompted the forecast of the “death of distance”^1^. However, even a most basic organization of society requires categories, compartments and borders to maintain order^2^. Confinement of human interactions to limited spatial areas is the key message of the long-standing hypothesis of Central Place Theory (CPT)^3^,^4^, which posits the existence of regular spatial patterns in regional human organization. In short, CPT assumes the existence of a “hierarchy” of regions that aims to explain the number, size and locations of human settlements with spatio-economic arguments. Despite its highly simplifying geometric assumptions (Supplementary Information), empirical evidence for CPT’s main prerequisite of systematically limited human interactions has been collected in a number of recent studies on massive interaction networks which have indeed observed a substantial impact of political or socio-economic boundaries on human interactions^5^,^6^,^7^,^8^,^9^,^10^,^11^. Typically, if we construct regions by clustering those locations that have strong interactions with each other, we divide countries into contiguous geographical regions with separating boundaries often following surprisingly close existing administrative boundaries. However, clustering is typically performed at the macroscopic level, in which nodes represent aggregated behavior of many users and the weight of the edges becomes the main structural element of the network^12^. As a consequence, the identified regions typically depict well-defined core areas corresponding to high-level centers of activity, like important cities, and their hinterlands (e.g. ref. 5 find 11 well-defined cores relating each to a densely populated area of Great Britain). These results, however, offer only a partial view, because human behavior, like communication and mobility, is nurtured by high-scale interactions and is increasingly becoming multi-scalar. This co-existence of short-range and long-range interactions^10^ raises the question whether high-level community detection offers a sufficient view for the development of models of human interaction. Should we rather look for an underlying, quantifiable principle that allows us to explain how high-level spatial regularities relate to local interactions that are at the same time short- and long-ranged? And if so, can we exploit this principle to develop better models of human interaction?

Despite the increasing availability of data and data-driven decision-making, modeling is important for several reasons. First, it is important to understand the underlying mechanisms behind human mobility and interactions for urban planning, to predict usage of future urban areas. Second, models are important for managing unforeseen events or major interventions on collective human behavior.

We start by introducing a quantitative metric, measuring the impact of borders on human interactions. For this purpose, we consider both mobile and landline phone communications. Next, we analyze the performance of state-of-the-art models that predict human interactions, revealing systematic biases in the way these models fit reality. These biases include the inability to capture the impact of borders and to reproduce important properties of the hierarchical structure of the human society. To solve these qualitative problems we propose a simple model that uses only a very course-grained knowledge of the country’s regional structure. Although our model clearly oversimplifies reality, it outperforms previous, more complex, models quantitatively, emphasizing the crucial nature of the impact of borders on human interactions.

## Results

### Quantifying the inhibitory effect of borders on human interaction

In order to quantify the hypothesized effect of hierarchical organization on human interactions, we first define consistent nested regional partitions by recursively applying the recently developed community detection algorithm “Combo”^13^ to country-wide phone call networks from the United Kingdom, Portugal, France, Ivory Coast and an anonymous country, Country X (Methods). As a modularity optimization heuristic, Combo might not always provide a global optimum solution, however it has been demonstrated to outperform the state-of-the-art community detection approaches providing the best known modularity score for many real-world and synthetic networks^13^. Although the data used in this study contains both mobile phone and landline phone call records, our results are qualitatively consistent and do not seem to depend on this circumstance, although a detailed comparison between the networks of human interactions inferred based on such two types of data might be a subject of an interesting separate study. Partitions resulting from this algorithm reflect the communities defined by underlying social interactions, and, contrary to official administrative boundaries, are independent of country-specific historical or political contexts^6^ (Supplementary Information). The resulting partition consists of three levels, *L*_1_, *L*_2_, and *L*_3_ which in general correspond to geographically cohesive regions, and are rather similar to administrative regions in number and size. As previously noted in refs 5, 6 (from which the partitions resulting from the Combo algorithm for UK and Portugal are further reproduced), these results may come as a surprise, as the modularity approach of the Combo algorithm has no spatial constraint nor does it impose any restriction on the number of communities.

**Figure 1:**
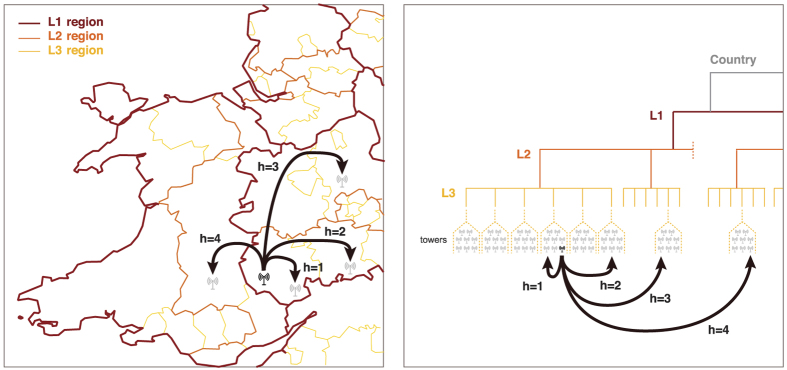
Partitioning of a country based on telephone call networks. Hierarchical distances between two locations are defined through three regional levels - either administrative ones or those found by applying iterative community detection on human interaction networks. Two distinct locations are at a hierarchical distance *h* = 1, if they are in the same *L*_3_ region, *h* = 2, if they are in different *L*_3_ regions but in the same *L*_2_ region, *h* = 3, if they are in different *L*_2_ regions but in the same *L*_1_ region and *h* = 4, if they are in different *L*_1_ regions. Note that a higher hierarchical distance does not necessarily correspond to higher geographical distance. The figure has been created using Matlab R2015b (http://www.mathworks.com) and publicly available shapefile data for the regional borders (http://ec.europa.eu/eurostat/web/gisco/geodata/reference-data/administrative-units-statistical-units, (c) EuroGeographics, 2016).

The above three levels have a natural interpretation: the whole country is divided into *L*_1_-level regions (regional scale), which are divided into *L*_2_-level regions (county scale) which in turn split into *L*_3_-level regions (city scale) composed of several “elementary” locations (cell phone tower or exchange area), Fig. 1. The number of levels is not imposed, but for all countries the process naturally stops subdividing regions at the city scale. Again we find that, Although no spatial constraints are applied, communities consist of contiguous locations at all levels. This observation has previously been reported just for *L*_1_-level regions using various data including phone call records^5^,^6^,^14^, vehicle GPS traces^15^, geo-tagged social media^16^ and credit card transactions^17^). We also find that the observed *L*_1_ regions are strikingly similar to administrative regions as highlighted by their comparison as well as by comparison with the random partitions (Supplementary Information, Table S4). This shows that current social interactions reflect most of the historic, political, infrastructural and other factors important for the administrative division of the country. However, the advanced question if and how the deviations between the *L*_1_ regions and the administrative boundaries can provide sufficient insights for adjustments or for considering additional factors, is a subject for further studies.

**Figure 2:**
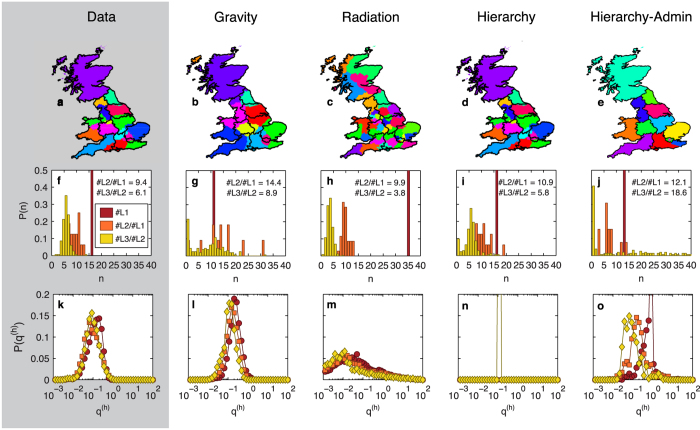
Hierarchical properties of spatial organization from human interactions. (**a–e)** Maps of *L*_1_ communities in telephone call networks detected from data and from various interaction models. Black lines correspond to the administrative partitioning of the 11 NUTS1 regions of UK, colored areas to regions detected by a community detection algorithm applied to (**a**) the data, and to the (**b**) gravity, (**c**) radiation, (**d**) hierarchy, and (**e**) administrative models. All detected regions are cohesive although some of the distinct colors used may appear similar. (**f**–**j**) Probability distribution of number of subregions by region found in (**f**) the actual network and (**g–j**) in each model. Averages corresponding to each distribution are indicated in each panel. This is a property that we expect the models to reproduce. The gravity model (**g**) underestimates the number of *L*_1_ communities but overestimates the numbers of subregions within regions. The radiation model (**h**) strongly overestimates the number of *L*_1_ communities. The hierarchy model (**i**) correctly determines the distributions of sub-communities per community. (**k**–**o**) Probability distributions of damping values *q*^(*h*)^ being an underlying property that is modeled in the hierarchy model (**n**) by a constant damping value for all levels. The distributions got from the data as well as those produced by the other models are also shown for the sake of comparison (although this is not the modeling objective). The figure has been created using Matlab R2015b (http://www.mathworks.com) and publicly available shapefile data for the regional borders (http://ec.europa.eu/euclidiantat/web/gisco/geodata/reference-data/administrative-units-statistical-units, (**c**) EuroGeographics, 2016).

Several insights that we first derive from these hierarchical partitions of empirical networks are in line with CPT. The *L*_3_ regions have typical spatial extension of a town with its neighborhood^18^ (between 15 km to 23 km, depending on the country). Similarly, *L*_2_ and *L*_1_ conform to the scales of districts and regions, respectively (Fig. 2a). The distribution *P(n*) of the number *n* of *L*_3_ communities inside a *L*_2_ community is strongly peaked around 6. This provides quantitative confirmation to the main hypothesis of regular spatial organization of CPT, which defines *K*-hexagonal landscapes (*K* = 3, 4, 6) as arrangements and each higher order settlement is supported by *K* − 1 lower order settlements and itself (see Supplementary information for more detail). Under this assumption, one would expect that each of the *L*_1_/*L*_2_ should consist of the same number of *L*_2_/*L*_3_ regions. Figure 2f shows that distribution, as well as the distribution of the number of *L*_2_ communities within an *L*_1_ community (#*L*2/#*L*1), for the UK. We observe similar peaks in all other countries (Figs S1–S4).

**Figure 3:**
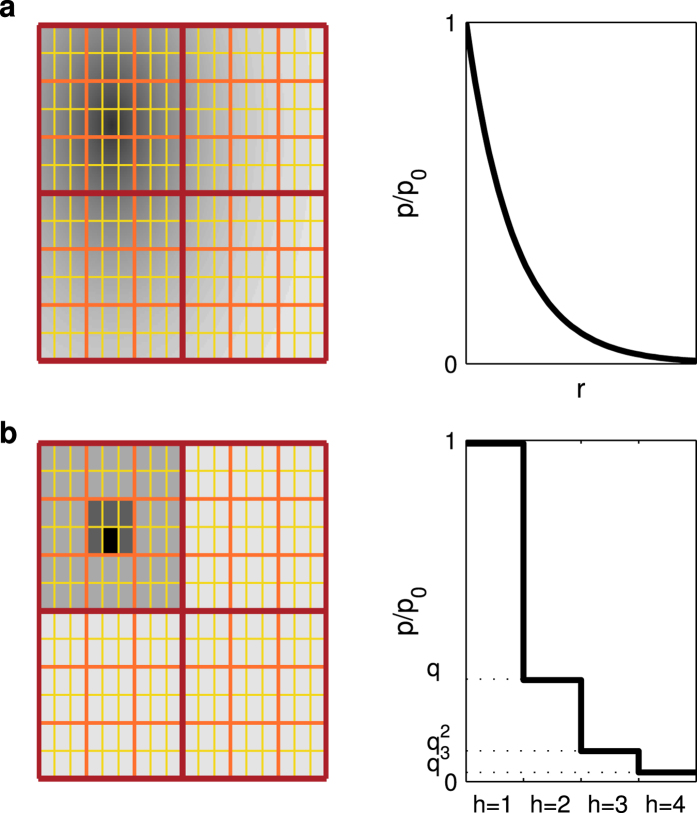
Schematic representation of the structural discontinuity effect. (**a**) In the classic gravity model, the probability *p* that two people communicate is a continuous (e.g. exponential) function of the distance between them. (**b**) In our hierarchy model, that probability is a discontinuous function induced by the assumption of a constant damping value *q* independent of the point of origin and the hierarchy level *h*. In both cases, the left panel shows in grayscale the probability of communication from a given point in space in a schematic country, partitioned in three regional levels with the same color coding as Fig. 1. The link between the borders and the deterrence function is clearly apparent in the second case.

Following the idea that borders inhibit human interaction, we introduce the notion of hierarchical distance to characterize its impact on communication flows (Fig. 1). Two locations *i* and *j* are at a hierarchical distance *h*_*ij*_ = 1, if they are in the same *L*_3_ region, at a distance *h*_*ij*_ = 2, if they are in different *L*_3_ regions, but in the same *L*_2_ region, at a distance *h*_*ij*_ = 3, if they are in different *L*_2_ regions, but in the same *L*_1_ region, or at a distance *h*_*ij*_ = 4, if they are in different *L*_1_ regions. In other words, the hierarchical distance corresponds to the number of different types of borders separating two locations. This metric only contains limited information about the spatial structure of the regions. It is only partly correlated with geographic distance: Two locations that are close in terms of geographic distance can still be situated in different *L*_1_ regions and hence far from each other in terms of hierarchical distance. Thus, the hierarchical distance is not a mere discretization of geographical distance, but encodes a qualitatively different, socio-economic notion of distance. To understand the impact of borders on human interaction on each hierarchical level, we define and measure the following damping parameters


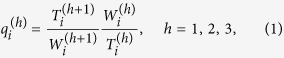


where 
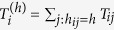
 is the total duration of calls originating from location *i* to all locations at hierarchical distance *h*. Defining the weight of node *i, w*_*i*_ = ∑_*j*_*T*_*ij*_, as the total duration of all calls originating from node *i* (including self-loops), 
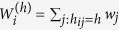
 is the total duration of all calls originating from locations at a hierarchical distance *h* from location *i*. The ratio 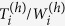 measures the relative strength of communication between location *i* and the locations at a hierarchical distance *h* from it. In particular, this ratio corresponds to the amount of communication sent to all locations at hierarchical distance *h* per unit of communication produced there. The damping value 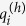, hence, measures the relative importance of locations at hierarchical distance *h* + 1 compared to those at hierarchical distance *h* from *i*. For example, we have 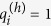 for all *h* means that communication from *i* is independent of the hierarchical distance, because there is no damping in the amount of communication sent per unit of communication produced as the hierarchical distance increases. Figure 2k shows the distributions of the damping values in the UK, all well peaked around a strikingly similar mean value. This result does not substantially change with the hierarchy level (*h* = 1, 2, 3), Table 1. Similar observations are made for all studied countries (Figs S1–S4). This finding reflects a structural discontinuity of human interactions and its consequences on modeling, see below, is our main discovery. It means that the damping effect of a boundary is approximately the same irrespective of the level *h* and origin location *i*, i.e. 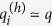. If the probability for two people who live in the same *L*_3_ region to communicate is *p*_0_, it will be *qp*_0_ for people living in different *L*_3_ regions but in the same *L*_2_ region, *q*^2^*p*_0_ if they live in the same *L*_1_ but different *L*_2_ region and *q*^3^*p*_0_ if they live in different *L*_1_ regions, Fig. 3b. Of course, reality has more gray-scale, just like the Fig. 3a does. However, we will see that even such an oversimplified assumption can still contribute quite a bit towards better understanding of that reality. In essence, structural discontinuity and hierarchical organization should be taken into account for a successful model of human interactions.

### Why and how standard models fail

Using several standard measures of fit statistic (the deviance, based on the log-likelihood, and other benchmark distances, see Methods) and comparing distributions of high level per low level regions, we test to which extent the most widely used models, namely gravity^19^ and radiation^20^, commit a systematic bias by failing to account for the observed boundary effects. To this end, we compute communication networks predicted by these models as well as the corresponding partitions resulting from the community detection algorithm (Methods).

**Figure 4:**
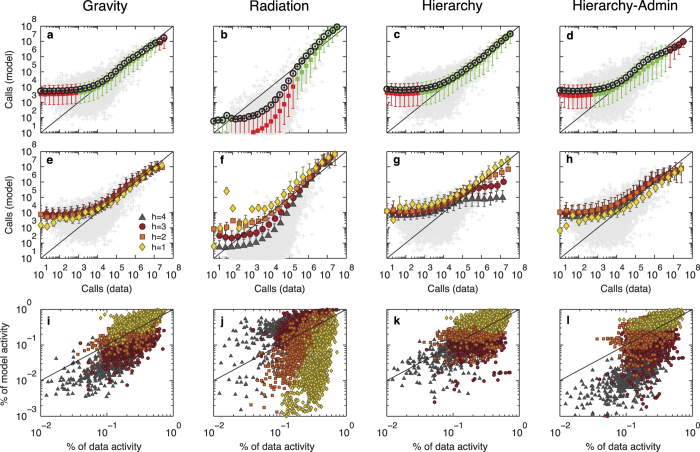
Comparison of model predictions. (**a–d**) Comparison of the actual total communication to the predicted communication for each pair of distinct locations, for the (**a**) gravity, (**b**) radiation, (**c**) hierarchy, and (**d**) administrative models. Gray markers are scatter plots for each pair of locations. A box is colored green if the equality line *y* = *x* lies between the 9th and 91th percentiles in that bin and is red otherwise. Red boxes hence emphasize significant biases of the models. Black circles correspond to the average total communication of the pairs of locations in that bin. **e–h**, Goodness of prediction with respect to the hierarchical distance *h*, for the (**e**) gravity, (**f**) radiation, (**g**) hierarchy, and (**h**) administrative models. Gray markers are scatter plots for each pair of locations. Error bars show the corresponding 9th and 91th percentiles of total communication values. (**i**–**l**) For each L3 community, comparison of the fractions of activity of model versus data between that L3 community and L3 communities at different hierarchical distances, for the (**i**) gravity, (**j**) radiation, (**k**) hierarchy and (**l**) administrative models.

As previously demonstrated^20^, the gravity model strongly underestimates and fails to predict high-range flows, i.e. flows between locations where the number of calls is high (Fig. 4a and Figs S5a to S8a). This certainly explains why the gravity model generates less and larger *L*_1_-regions and why their subdivisions do not follow the narrow distributions observed in the data (Fig. 2b,g). The damping value predicted by the gravity model is otherwise well peaked, although its average values vary from one *h*-level to another (Table 1).

In contrast, the radiation model overestimates long-range flows (Fig. 4b), resulting in more and smaller *L*_1_-regions (Fig. 2c). The distribution of *L*_3_ within *L*_2_ regions is still well-peaked, but shifted to the left (Fig. 2h). Moreover, the distribution of damping values in the radiation model is strongly spread out (Fig. 2m and Table 1), and does not reproduce the existence of a single typical damping parameter. Similar systematic biases of gravity and radiation models become evident, if we measure the probability *P*_dist_(*d*) of a call between locations at distance *d* (Fig. S9).

The poorer current performance of the radiation model compared to the examples of the original paper^20^ originates in part from to the different type of spatial flows considered. In fact, while in ref. 20 the flows were defined as the number of calls made by users resident in different municipalities, in the present paper the flows correspond to the total duration of calls between the current locations of the callers and callees at the moment of the calls. These differences affect the communication network in two main ways. First, in the present paper the number of calls is weighted by their duration, so for example one 10-minute long-distance call is equivalent to ten 1-minute short-distance calls, whereas in ref. 20 only the number of calls was considered. Second, in the present paper we consider the distance between the locations of the caller and callee at the moment of the call. If, for instance, an individual who is currently on a business trip in a city at 500 km from her/his home location calls a family member at home, this generates a long-distance flow between the two locations. In ref. 20 this would generate a short-distance flow within the same home location, as only the individuals’ resident locations are considered. As a result, in this paper we observe many more long-distance flows (see Fig. S9) than in ref. 20. This effect is not accounted for by the original radiation model. However, as demonstrated in the SI (Tables S5 and S6), the generalized version of the radiation model proposed in ref. 21, which depends on one free parameter adjusting the median distance of the flows, has a performance comparable and in some cases superior to the other models. This suggests that the radiation model is able to accurately estimate the flows when the spatial scale is properly adjusted.

### Accounting for strong border effects with the Hierarchy model

The two most commonly used models thus fail to reproduce the boundary effect. By design, a model taking into account the observed hierarchical structures by assuming a constant damping value *q*, would overcome this issue (Fig. 3b). Consider the minimal model in the stylized form 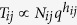, where *N*_*ij*_ represents the potential pairs of contacts between two distinct locations *i* and *j* and 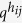 denotes the probability for two people from these locations to communicate. This model would implement highly discretized hierarchical distances instead of considering a continuum of geographical distances. Similarly to the gravity model, *N*_*ij*_ can be taken as proportional to the weights *w*_*i*_ and *w*_*j*_ of both origin and destination locations. We therefore propose a simple *hierarchy model* that predicts an interaction strength as


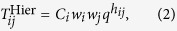


a power-law form, where 0 < *q* < 1 is a parameter to be determined and *C*_*i*_ are local normalization factors ensuring 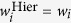. This normalization also ensures that the damping values are constants, 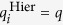 (see proof in Supplementary Information). The best-fit values of *q* are very close to the observed values (Table 1 and Supplementary Information, Table S1) and robust to small variation (Fig. S10). They slightly depend on the country, varying between 0.10 and 0.25, reflecting differences in the structural properties of the studied networks. The hierarchy model reproduces almost perfectly the nested structure of regions (Fig. 2d,i), while the distribution of damping values stays as imposed (Fig. 2n). To our surprise, the hierarchy model also outperforms the state-of-the-art models in terms of goodness of fit measures (Table 2 and Supplementary Information, Table S2). In particular, it estimates high-range flows with a greater accuracy than the radiation or gravity models, as can be seen on the top right corners of Fig. 4c and Figs S5c to S8c, where the markers are typically closer to the equality line than in state-of-the-art models.

## Discussion

While modeling human interactions over space we have introduced a concept of a hierarchical distance and a new model based on it. We have first focused on the flows between locations at specific hierarchical distance. The goodness of prediction of the different models vs the ground truth has provided by both cellular and landline phone data is informative to understand why the proposed hierarchy model outperforms the others. The radiation model overestimates the flows at *h* = 1, 2, the corresponding markers being above the equality line in Fig. 3f and Figs S5f to S8f resulting in an overall overestimation quantified by values of *R*_*h*=1,2_ (Methods) greater than 1 (Table 3), and underestimates those at *h* = 3, 4. On the contrary, the gravity model underestimates the flows at *h* = 1, 2 and overestimates those at *h* = 3, 4 (Table 3, Fig. 4e, Figs S5e to S8e). This also results is an overall bias of the models on the inter-regional level (Fig. 4i,j). The hierarchy model produces more balanced predictions (*R*_*h*_ closer to 1; see Fig. 4g,k) and thus outperforms existing models.

The hierarchy model requires the knowledge of the communication flows in order to determine the three hierarchical levels each location belongs to. However, it can also be applied in the absence of communication data, using the administrative boundaries and a general damping value *q* = 0.2. This pre-determines the model’s ability to reproduce the properties of the nested structure of human society (Fig. 2e,j,o) for all the countries (Supplementary Information, Figs S1–3). The resulting *hierarchy-admin* model based on this administrative partition is parameter-free. Yet it provides similar or sometimes better estimates than the gravity model in terms of communication flow (Fig. 4d,h,l, Figs S5k to S8k: See in particular the case of high-range flow in Portugal and Ivory Coast) or benchmark measures (Table 2 and Supplementary Information, Table S2). We have also tested different constraint conditions and deterrence functions *f* in the hierarchy model 
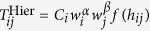
. We have compared them to multiple variations of the gravity and radiation models and found that they are widely outperformed by hierarchy models (Supplementary Information, Tables S5 and S6).

## Conclusion

In summary, we have first defined communication flows-induced boundaries by applying standard community detection methods on large-scale human interaction networks and found that these networks have a nested structure reflecting historic, socio-political borders. This can be related to the structure predicted by CPT. We introduced the notion of damping parameter that represents the normalized ratio of interactions between locations at different hierarchical distances. This has enabled us to quantify the inhibiting effect of boundaries. Surprisingly, the distributions of damping parameters are well-peaked and largely independent of the hierarchical level, revealing a structural discontinuity effect in every country considered. We have further shown that current models of human interaction, which are based only on population and/or geographical distance, cannot correctly reproduce the characteristic hierarchical structure of interaction networks. We have proposed a simple model based on the discrete hierarchical distance that outperforms the state-of-the-art models of human interaction in a number of different countries. This demonstrates its general applicability and emphasizes the impact of the borders on human interactions.

One can notice, however, that the model clearly oversimplifies the reality. While we find that the impact of borders dominate over the impact of geographical distance, it would be reasonable to assume that distance still matters for pairs of locations within the same hierarchical distance. For the purpose of this present study, we have intentionally kept the hierarchical model as simple as possible in order to clearly emphasize the isolated impact of society’s hierarchical structure. However, development of more sophisticated models combining both geographic and socio-political information can further boost our ability to understand and reproduce the structure of social systems.

Another potential future development for the model might include an optimization of the hierarchy and the hierarchical distance to be the most consistent with the observed structure of human interactions, i.e. optimizing the fit of the corresponding model based on it. Considering different hierarchical distances - like the one based on existing administrative boundaries and the one produced by community detection - can largely impact the model performance. This could point the way for an approach to define the optimal administrative boundaries and could have modeling implication based on both social connections as well as the spatial layout alternatively to the network community detection approach considered in the present paper.

In any case we believe that the present research highlights the importance and the impact of regional borders to be considered as a vital ingredient for modeling human interactions and/or mobility – an ingredient that seems to have been missing so far.

## Methods

### Telephone call data

We consider several country-wide data sets of telephone calls, including the four European countries of the UK^5^,^6^, France^6^,^22^,^23^, Portugal^6^,^23^,^24^,^25^, an anonymized Country X^23^, and Ivory Coast^6^,^8^,^26^ (the references point to publications where the corresponding datasets have been used previously). All data sets comprise mobile phone data with the exception of landline calls in the UK. Data was provided by single phone providers with possibly heterogeneous coverage over the respective countries. We have no information on local market shares and on resulting possible inhomogeneities in spatial coverage. Specific details of the different datasets are provided in Table 4, all of them gathering millions of users making billions of calls during time frames ranging from 1 to 15 months.

The Ivory Coast data was released to researchers during the D4D mobile phone data challenge^27^ and was used as is. Researchers interested in getting access to this dataset might reach out to the D4D challenge organizers. All other data sets are proprietary and subject to stricter data non-disclosure agreements. Therefore, we do not have the possibility to share the raw data nor to provide more expressive information on metadata or on the data collection process available than provided in Table 4. All data has been anonymized and/or aggregated on the operator side prior to receipt and in line with all local data protection laws.

We construct interaction networks between different locations of a country based on the aggregated duration of calls having origin in the first and destination in the second location. This process generates weighted directed networks in which loop edges from locations to themselves are also considered, and where the link weight *T*_*ij*_ between a location *i* and location *j* is defined as the total duration (or, in case of Country X, total number) of calls from location *i* to location *j*. The nodes of the network are the locations, corresponding to exchange areas or cell towers areas as reported in Table 4. In all datasets, the users are attached to the actual locations where the calls occur, i.e. not necessarily their residential locations. In case of mobile phone connections, each call contributes to the link between the current location of the caller and the location of the recipient as of the moment of the call).

### Network partitioning

A recently developed algorithm for community detection, referred to as “Combo”^5^,^6^,^8^, is applied to the extracted communication networks to detect communities of highly connected locations. The method follows a standard modularity optimization approach^28^,^29^. It scores the edges of the networks according to their relative strength compared to a null-model based on the weight of the nodes they connect and aims at maximizing the cumulative score inside the communities. Given a partition of the nodes in a set of clusters *c*_*i*_, the modularity score *Q* is given by


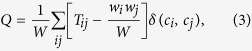


where *T*_*ij*_ is the weight of the link between node *i* and node *j, w*_*i*_ = ∑_*j*_*T*_*ij*_ is the weight of node *i* and *W* = ∑_*i*_*w*_*i*_/2 is the total weight of the network. While the outcome of partitioning is in general not qualitatively dependent on the particular algorithm used, the Combo algorithm has the ability to consistently provide the best results in terms of modularity score compared to other algorithms^13^. The modularity optimization approach yields communities whose size and properties are only based on the information of the links’ weights. See ref. 30 for a more explicit interpretation of the modularity, its properties and limits.

Applying the Combo algorithm yields a first partition of the network into communities, further referred to as “level 1” or “*L*_1_” partition. To obtain the substructure of these communities, we iteratively apply the Combo algorithm on each *L*_1_ community, thus creating a“level 2” or “*L*_2_” community partition, and and then again on each *L*_2_ community, thus creating a “level 3” or “*L*_3_” community partition. We find that most of the *L*_1_ and *L*_2_ communities display a clear substructure with high values of internal modularity scores, typically around 0.4 and 0.7 (Supplementary Information, Table S4). The resulting communities consists in geographically cohesive regions, which can seem surprising since the algorithm uses only the networks topology and no geographical information, such as the distance between the nodes (Supplementary Information). This cohesiveness is also linked to the spatial scale of the studied network: We would not expect any contiguous communities, if that analysis was done at a city scale, where the movements and communications of individuals are more evenly distributed in space.

### Interactions models and goodness measures

The radiation model is a parameter-free model recently introduced in the context of migration patterns^20^. Given the geographic distance *d*_*ij*_ between two locations *i* and *j*, the model predicts that the flow of individuals moves *T*_*ij*_ between these two distinct locations will depend on the population at the origin, the population at the destination and on the population *s*_*ij*_ within the circle of radius *d*_*ij*_ centered on the origin location *i*. Applied to our case (using the total communication *w*_*i*_ at location *i* as a proxy for its population), the radiation model can be written as


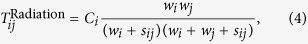


where 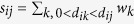 is the total amount of communication originating from locations at a distance shorter than *d*_*ij*_ from location *i* and *C*_*i*_ is a normalization factor ensuring that the predicted total activity of each node is the same than the actual one, i.e. 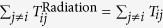. The model is otherwise parameter-free.

The gravity model is one of the oldest models describing human mobility and interaction, formulated in analogy to Newton’s law of gravity. The classical form predicts that the interaction strength between two distinct locations varies with the distance between them according to a power law:


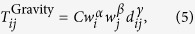


where *C* is a global normalization constant ensuring that 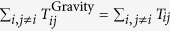 and *α, β*, and *γ* are parameters to fit.

We also computed the generalized version of the radiation model proposed in ref. 21, as well as different versions of the gravity and hierarchy models, comparing the results obtained using a power-law or exponential deterrence function (Supplementary Information). All parameters in these models were estimated through a regression analysis minimizing the deviance *E*^31^, a measure based on the log-likelihood of model compared to a saturated model that can be interpreted as a generalization of the residual sum of squares *R*^2^. While a fair comparison between the models also requires to take the variable number of parameters into account, the deviance *E* is related to the Akaike Information Criterion *AIC*, a criterion used to compare models with different numbers of parameters *k*, by *AIC* = 2*k* + *E*. In our cases, *k* = 0, 1 or 3 and 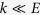, hence *AIC* ~ *E*.

We also quantify the fits between communication networks and models through different benchmark measures, namely the Dice distance *D*, the Sorensen distance *S*, and the cosine distance *C* defined by:


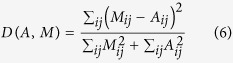



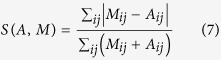



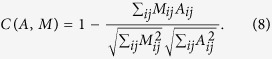


These three benchmark measures cover most families of distance measures^32^, which allows us to ensure that our findings are stable with respect to the distance measure used. They all vary between 0 and 1 and the lower they are, the more similar the model is to the original data.

Finally, we also computed the correlation *corr* between each model and the data defined by


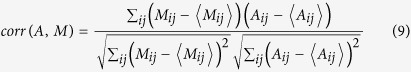


which is a measure of similarity varying between −1 and 1 (the closer to 1, the higher the similarity).

### Over- and underestimation measure

In order to determine whether a given subset of links are over- or underestimated by the models, we define for any given set *E* of links, the following ratio:


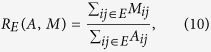


where we use the notation *A* for the data and *M* for the model. Values of *R*_*E*_ larger (smaller) than 1 hence correspond to an overestimation (underestimation) of the model. The measure *R*_*E*_ provides an aggregated knowledge dominated by link weights.

## Additional Information

**How to cite this article:** Grauwin, S. *et al*. Identifying and modeling the structural discontinuities of human interactions. *Sci. Rep.* **7**, 46677; doi: 10.1038/srep46677 (2017).

**Publisher's note:** Springer Nature remains neutral with regard to jurisdictional claims in published maps and institutional affiliations.

## Supplementary Material

Supplementary Information

## Figures and Tables

**Table 1:** Values of the damping value *q* for the actual and modeled networks in the UK.

Data set/Network	〈*q*^(1)^〉	〈*q*^(2)^〉	〈*q*^(3)^〉
Data	0.180 ± 0.002	0.143 ± 0.002	0.144 ± 0.002
Gravity	0.331 ± 0.005	0.234 ± 0.003	0.167 ± 0.002
Radiation	8.180 ± 6.039	6.156 ± 3.922	3.753 ± 1.687
Hierarchy	0.139 ± 0.000	0.139 ± 0.000	0.139 ± 0.000
Hierarchy-Admin	0.2 ± 0.0	0.2 ± 0.0	0.2 ± 0.0

**Table 2:** Benchmark measures quantifying the goodness of fit in the UK.

Model	E × 10^−12^	D	S	C	corr	parameters
Gravity	0.494	0.456	0.448	0.456	0.543	*α* = 0.65, *β* = 0.65, *γ* = −1.46
Radiation	1.622	0.624	0.632	0.344	0.656	
Hierarchy	0.464	0.233	0.437	0.231	0.768	*q* = 0.139
Hierarchy-Admin	0.679	0.503	0.527	0.458	0.540	*q* = 0.2 (imposed)

The Dice (D), Sorensen (S), Cosine (C) and deviance (E) are four different measures of the distance between the actual and modeled networks. The correlation *corr* measures a similarity between a model and the data. The parameters of the gravity and hierarchy models were chosen to minimize the value of E.

**Table 3:** Over-/under-estimation measures of link at specific hierarchical distance in the UK.

Model	*R*_*h* = 1_	*R*_*h* = 2_	*R*_*h* = 3_	*R*_*h* = 4_
Gravity	0.54	0.73	1.15	1.33
Radiation	2.39	1.47	0.67	0.16
Hierarchy	1.10	0.73	0.90	1.18
Hierarchy-Admin	0.25	0.73	1.43	1.30

**Table 4:** Properties of the data sets.

Data set	Calls	Duration (s)	Phones	Time	Spatial resolution	*ρ*_*links*_	Directed
France	218 m.	47 bn.	17.6 m.	1 month	8,800 areas	11.6%	yes
UK	7.6 bn.	452 bn.	47 m.	1 month	4,800 areas	37.6%	no
Portugal	440 m.	56 bn.	1.6 m.	15 months	2,200 cell towers	83.1%	yes
Country X	1.1 bn.	-	6.9 m.	12 months	9,400 cell towers	28.0%	yes
Ivory Coast	62 m.	7 bn.	5 m.	6 months	1,250 cell towers	84.2%	no

Country-wide telephone data sets are provided by single telephone operators, covering different time frames, with different numbers of phones, calls, total call durations and on various spatial resolutions. The abbreviations bn. and m. stand for billion and million, respectively. Resolution numbers are given as approximate values. These locations constitute the nodes of the corresponding telephone call networks, while the sum of durations of calls between locations span their weighted links. The last columns report the percentage of non-zero links between pairs of nodes in the extracted network and whether or not that network is directed. The durations of calls are unknown in the case of Country X. All datasets corresponds to mobile phone network except for UK, where the dataset corresponds to a landline network.
