# Supplementary material for: Identifying and modeling the structural discontinuities of human interactions
Source: Sci Rep. 2017 Apr 26;7:46677. doi: 10.1038/srep46677 (PMC5405407; doi:10.1038/srep46677)
Supplement: Supplementary Information [file srep46677-s1.pdf]

Identifying and modeling the structural discontinuities of human interactions - Supplementary Information

Sebastian Grauwin[1], Michael Szell[1,2,3], Stanislav Sobolevsky[1,4,*], Philipp Hövel[2,5,6], Filippo Simini[2,7,8], Maarten Vanhoof[9], Zbigniew Smoreda[9], Albert-László Barabási[2,10,11], and Carlo Ratti[1]

[1]Senseable City Lab, Massachusetts Institute of Technology, 77 Massachusetts Avenue, Cambridge, MA 02139, USA
[2]Center for Complex Network Research, Northeastern University, 110 Forsyth Street, Boston, MA 02115, USA
[3]Hungarian Academy of Sciences, Centre for Social Sciences, Tóth Kálmán utca 4, 1097 Budapest, Hungary
[4]Center for Urban Science And Progress, New York University, 1 MetroTech Center, 19fl, Brooklyn, NY 11201, USA
[5]Institut für Theoretische Physik, Technische Universität Berlin, Hardenbergstraße 36, 10623 Berlin, Germany
[6]Bernstein Center for Computational Neuroscience, Humboldt-Universität zu Berlin, Philippstraße 13, 10115 Berlin, Germany
[7]Department of Engineering Mathematics, University of Bristol, Woodland Road, Bristol, BS8 1UB, United Kingdom
[8]Institute of Physics, Budapest University of Technology and Economics, Budafoki út 8, Budapest, H-1111, Hungary
[9]Département SENSE, Orange Labs, 38 rue du Général Leclerc, 92794 Issy-les-Moulineaux, France
[10]Center for Cancer Systems Biology, Dana-Farber Cancer Institute, Boston, Massachusetts 02115, USA
[11]Department of Medicine, Brigham and Women's Hospital, Harvard Medical School, Boston, Massachusetts 02115, USA

ABSTRACT

The idea of a hierarchical spatial organization of society lies at the core of seminal theories in human geography that have strongly influenced our understanding of social organization. In the same line, the recent availability of large-scale human mobility and communication data has offered novel quantitative insights hinting at a strong geographical confinement of human interactions within neighboring regions, extending to local levels within countries. However, models of human interaction largely ignore this effect. Here, we analyze several country-wide networks of telephone calls - both, mobile and landline - and in either case uncover a systematic decrease of communication induced by borders which we identify as the missing variable in state-of-the-art models. Using this empirical evidence, we propose an alternative modeling framework that naturally stylize the damping effect of borders. We show that this new notion substantially improves the predictive power of widely used interaction models, thus increasing our ability to understand, model and predict social activities and to plan the development of infrastructures across multiple scales.

Supplementary Tables

Table S1. Values of the damping parameter q for the actual and modeled networks in France, Portugal, Country X and Ivory Coast.

Data set / Network	$\langle q^{(1)} \rangle$	$\langle q^{(2)} \rangle$	$\langle q^{(3)} \rangle$
UK / Data	0.180±0.002	0.143±0.002	0.144±0.002
UK / Gravity	0.331±0.005	0.234±0.003	0.167±0.002
UK / Radiation	8.180±6.039	6.156±3.922	3.753±1.687
UK / Hierarchy	0.139±0.000	0.139±0.000	0.139±0.000
UK / Hierarchy-Admin	0.2±0.0	0.2±0.0	0.2±0.0
Portugal / Data	0.324±0.032	0.331±0.006	0.286±0.005
Portugal / Radiation	4.639±1.881	6.759±2.254	198.3±186.4
Portugal / Gravity	0.487±0.004	0.527±0.005	0.377±0.003
Portugal / Hierarchy	0.258±0.000	0.258±0.000	0.258±0.000
Portugal / Hierarchy-Admin	0.200±0.000	0.200±0.000	0.200±0.000
France / Data	0.196±0.007	0.290±0.081	0.154±0.004
France / Radiation	13.60±5.42	656.8±507.2	25648±24798
France / Gravity	0.287±0.003	0.263±0.002	0.166±0.002
France / Hierarchy	0.158±0.000	0.158±0.000	0.158±0.000
France / Hierarchy-Admin	0.200±0.000	0.200±0.000	0.200±0.000
Country X / Data	0.237±0.002	0.168±0.002	0.056±0.001
Country X / Radiation	38.52±19.41	9439±6096	288.4±189.2
Country X / Gravity	0.329±0.005	0.286±0.009	0.135±0.001
Country X / Hierarchy	0.114±0.000	0.114±0.000	0.114± 0.000
Country X / Hierarchy-Admin	0.200±0.000	0.200±0.000	0.200±0.000
Ivory Coast / Data	0.324±0.005	0.251±0.005	0.262±0.005
Ivory Coast / Radiation	9.465±4.055	5.475±2.664	3.328±1.404
Ivory Coast / Gravity	0.619±0.006	0.577±0.005	0.489±0.004
Ivory Coast / Hierarchy	0.255±0.000	0.255±0.000	0.255±0.000
Ivory Coast / Hierarchy-Admin	0.200±0.000	0.200±0.000	0.200±0.000

Table S2. Benchmark measures quantifying the goodness of fit in Portugal, France, Country X and Ivory Coast. The Dice (D), Sorensen (S), Cosine (C) and deviance (E) are four different measures of the distance between the actual and modeled networks. The correlation *corr* measures a similarity between a model and the data. The parameters of the gravity and hierarchy models were chosen to minimize the value of E.

Country / Model	$E\times10^{-9}$	D	S	C	corr	fitted parameters
Portugal / Radiation	314.1	0.781	0.739	0.476	0.525	
Portugal / Gravity	79.80	0.865	0.419	0.844	0.145	$\alpha = 0.81$, $\beta = 0.79$, $\gamma = -0.71$
Portugal / Hierarchy	66.66	0.346	0.404	0.308	0.683	$q = 0.258$
Portugal / Hierarchy-Admin	74.20	0.456	0.416	0.362	0.627	$q = 0.278$
France / Radiation	227.758	0.618	0.647	0.270	0.730	
France / Gravity	90.905	0.267	0.524	0.185	0.815	$\alpha = 0.69$, $\beta = 0.69$, $\gamma = -1.44$
France / Hierarchy	73.524	0.341	0.514	0.267	0.733	$q = 0.158$
France / Hierarchy-Admin	80.686	0.212	0.529	0.207	0.793	$q = 0.192$
Country X / Radiation	3.701	0.577	0.638	0.356	0.644	
Country X / Gravity	1.483	0.472	0.467	0.470	0.529	$\alpha = 0.81$, $\beta = 0.78$, $\gamma = -1.06$
Country X / Hierarchy	1.120	0.255	0.456	0.252	0.748	$q = 0.114$
Country X / Hierarchy-Admin	2.076	0.743	0.547	0.565	0.434	$q = 0.158$
Ivory Coast / Radiation	268.18	0.701	0.703	0.358	0.645	
Ivory Coast / Gravity	68.17	0.577	0.413	0.460	0.519	$\alpha = 0.94$, $\beta = 0.94$, $\gamma = -0.51$
Ivory Coast / Hierarchy	42.90	0.228	0.351	0.217	0.775	$q = 0.255$
Ivory Coast / Hierarchy-Admin	65.98	0.437	0.430	0.309	0.681	$q = 0.394$

Table S3. **Over- / under-estimation measures of link at specific hierarchical distance in France, Portugal, Country X and Ivory Coast.**

Country / Model	$R_{h=1}$	$R_{h=2}$	$R_{h=3}$	$R_{h=4}$
UK / Gravity	0.54	0.73	1.15	1.33
UK / Radiation	2.39	1.47	0.67	0.16
UK / Hierarchy	1.10	0.73	0.90	1.18
UK / Hierarchy-Admin	0.25	0.73	1.43	1.30
Portugal / Radiation	3.95	1.02	0.33	0.08
Portugal / Gravity	0.54	0.75	1.09	1.27
Portugal / Hierarchy	1.10	0.95	0.85	1.10
Portugal / Hierarchy-Admin	0.70	1.14	1.17	0.93
France / Radiation	2.56	1.12	0.42	0.10
France / Gravity	0.60	0.91	1.31	1.16
France / Hierarchy	0.91	0.75	0.88	1.34
France / Hierarchy-Admin	0.42	0.98	0.84	1.58
Country X / Radiation	2.53	0.76	0.28	0.11
Country X / Gravity	0.81	0.94	1.03	0.52
Country X / Hierarchy	1.25	0.59	0.58	2.09
Country X / Radiation	3.95	1.02	0.33	0.08
Country X/ Hierarchy-Admin	0.12	0.43	1.59	2.60
Ivory Coast / Radiation	4.51	1.79	0.63	0.12
Ivory Coast / Gravity	0.29	0.47	1.02	1.33
Ivory Coast / Hierarchy	1.18	0.86	0.94	1.03
Ivory Coast / Hierarchy-Admin	0.74	1.03	1.13	0.99

Supplementary Figures

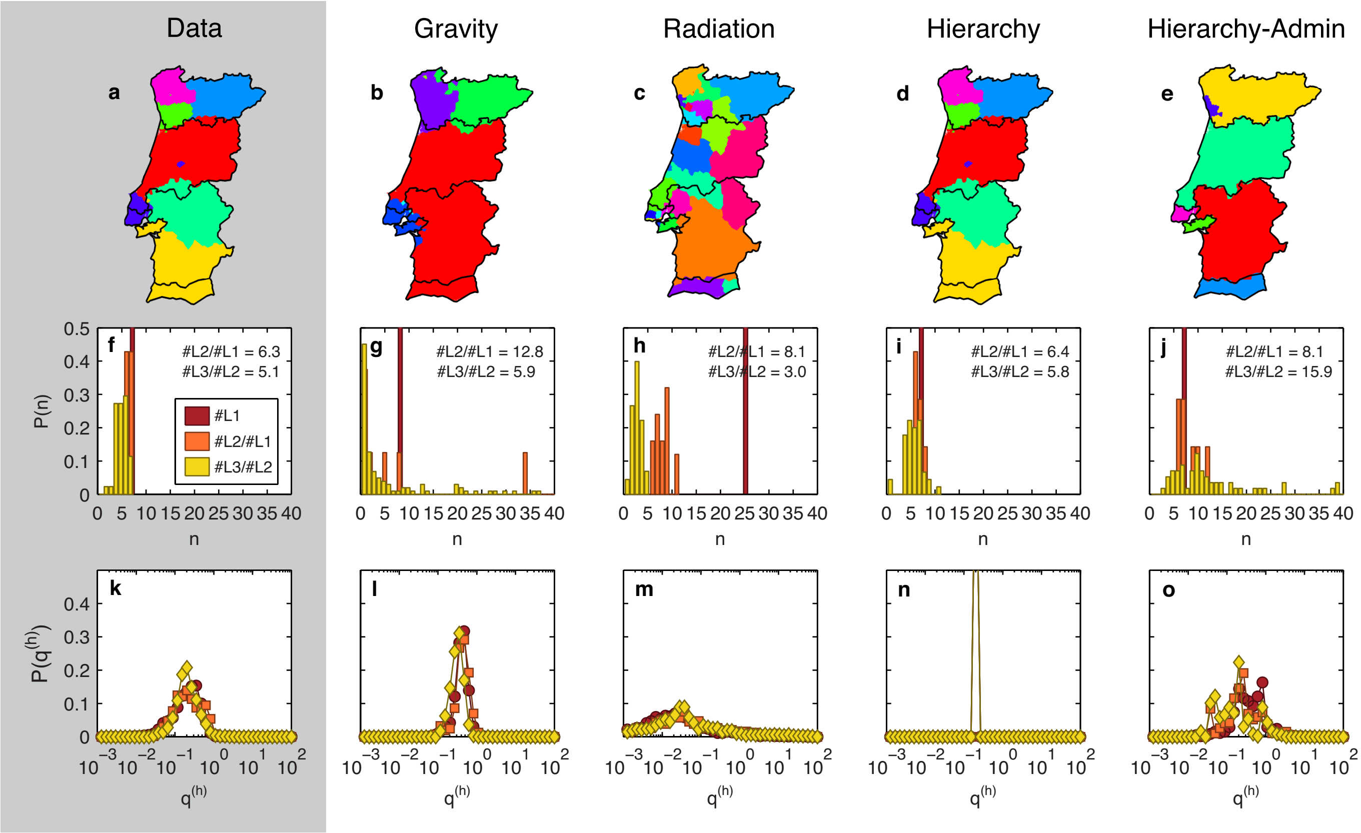

Figure S1. **Hierarchical properties of spatial organization from human interactions in Portugal.** **a–e**, Maps of L_1 communities in telephone call networks detected from data and from various interaction models. Black lines correspond to the administrative partitioning of the 5 NUTS1 regions of Portugal, colored areas to regions detected by a community detection algorithm applied to (**a**) the data, and to the (**b**) gravity, (**c**) radiation, (**d**) hierarchy, and (**e**) administrative models. All detected regions are cohesive although some of the distinct colors used may appear similar. **f–j**, Probability distribution of number of subregions by region found in (**f**) the actual network and (**g–j**) in each model. The gravity model (**g**) underestimates the number of L_1 communities but overestimates the numbers of subregions within regions. The radiation model (**h**) strongly overestimates the number of L_1 communities. The hierarchy model (**i**) correctly determines the distributions of sub-communities per community. **k–o**, Probability distributions of damping values $q^{(h)}$. The hierarchy model (**n**) assumes a constant damping value for all levels. The figure has been created using Matlab R2015b (http://www.mathworks.com) and publicly available shapefile data for the regional borders (http://ec.europa.eu/eurostat/web/gisco/geodata/reference-data/administrative-units-statistical-units, (c) EuroGeographics, 2016).

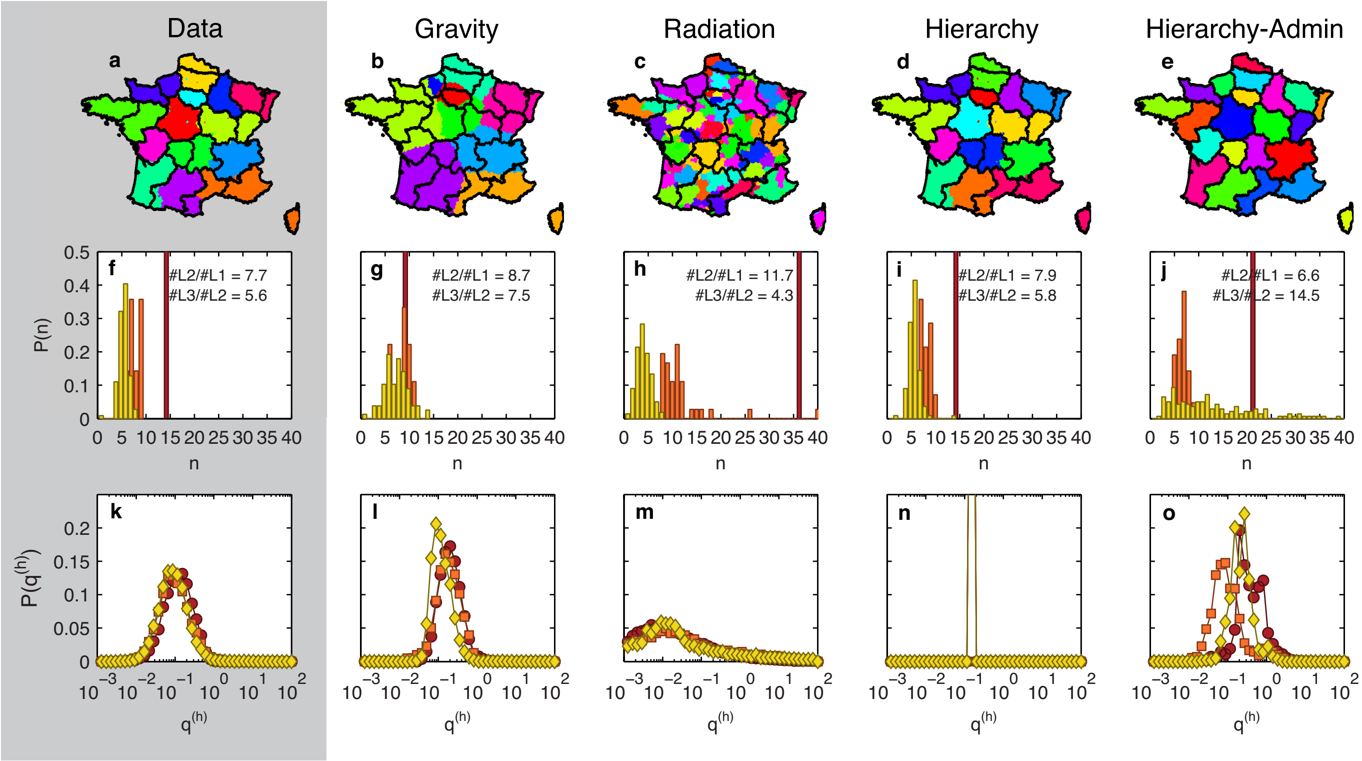

Figure S2. **Hierarchical properties of spatial organization from human interactions in France. a–e**, Maps of L_1 communities in telephone call networks detected from data and from various interaction models. Black lines correspond to the administrative partitioning of the 22 NUTS1 regions of France, colored areas to regions detected by a community detection algorithm applied to (**a**) the data, and to the (**b**) gravity, (**c**) radiation, (**d**) hierarchy, and (**e**) administrative models. All detected regions are cohesive although some of the distinct colors used may appear similar. **f–j**, Probability distribution of number of subregions by region found in (**f**) the actual network and (**g–j**) in each model. The gravity model (**g**) underestimates the number of L_1 communities but overestimates the numbers of subregions within regions. The radiation model (**h**) strongly overestimates the number of L_1 communities. The hierarchy model (**i**) correctly determines the distributions of sub-communities per community. **k–o**, Probability distributions of damping values $q^{(h)}$. The hierarchy model (**n**) assumes a constant damping value for all levels. The figure has been created using Matlab R2015b (http://www.mathworks.com) and publicly available shapefile data for the regional borders (http://ec.europa.eu/eurostat/web/gisco/geodata/reference-data/administrative-units-statistical-units, (c) EuroGeographics, 2016).

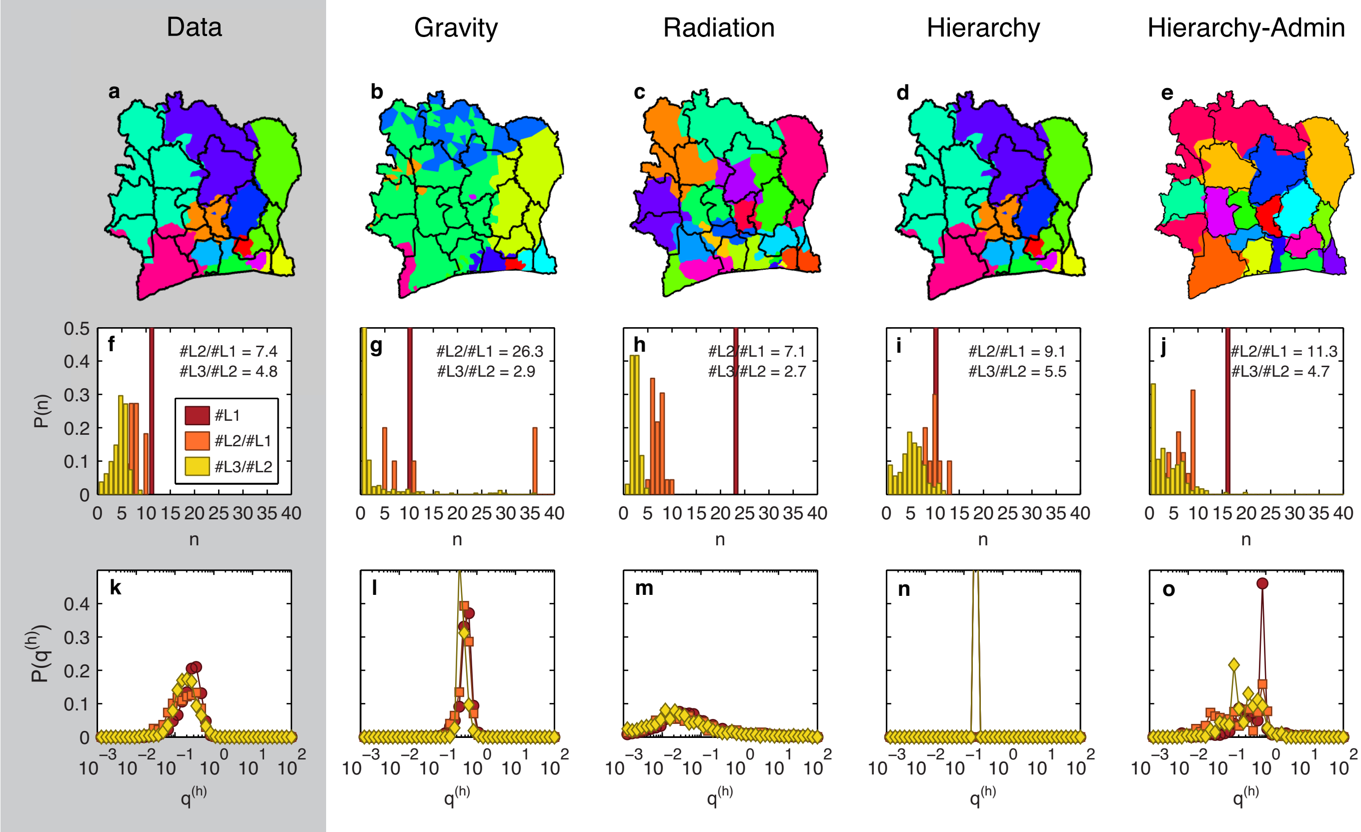

Figure S3. **Hierarchical properties of spatial organization from human interactions in Ivory Coast. a–e**, Maps of L_1 communities in telephone call networks detected from data and from various interaction models. Black lines correspond to the administrative partitioning of the 19 administrative regions of Ivory Coast, colored areas to regions detected by a community detection algorithm applied to (**a**) the data, and to the (**b**) gravity, (**c**) radiation, (**d**) hierarchy, and (**e**) administrative models. All detected regions are cohesive although some of the distinct colors used may appear similar. **f–j**, Probability distribution of number of subregions by region found in (**f**) the actual network and (**g–j**) in each model. The gravity model (**g**) underestimates the number of L_1 communities but overestimates the numbers of subregions within regions. The radiation model (**h**) strongly overestimates the number of L_1 communities. The hierarchy model (**i**) correctly determines the distributions of sub-communities per community. **k–o**, Probability distributions of damping values $q^{(h)}$. The hierarchy model (**n**) assumes a constant damping value for all levels. The figure has been created using Matlab R2015b (http://www.mathworks.com) and publicly available shapefile data for the regional borders (http://ec.europa.eu/eurostat/web/gisco/geodata/reference-data/administrative-units-statistical-units, (c) EuroGeographics, 2016).

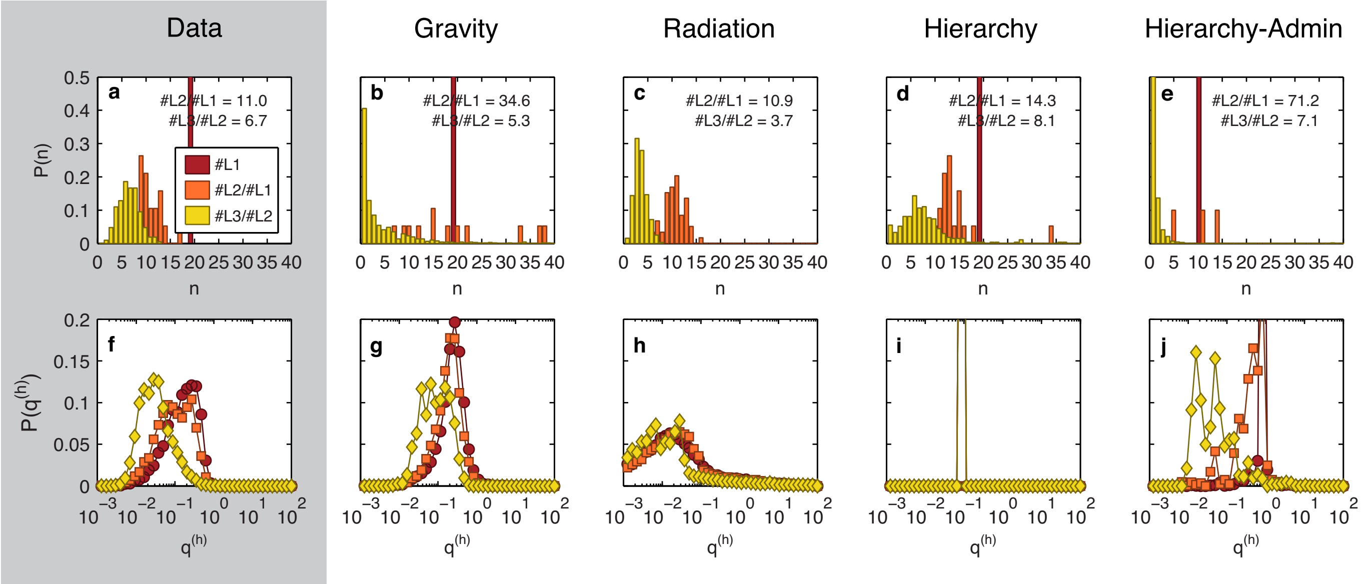

Figure S4. **Hierarchical properties of spatial organization from human interactions in Country X. a–e**, Probability distribution of number of subregions by region of Country X found in (**a**) the actual network and (**b–e**) in each model. The gravity model (**b**) underestimates the number of L_1 communities but overestimates the numbers of subregions within regions. The radiation model (**c**) strongly overestimates the number of L_1 communities. The hierarchy model (**d**) correctly determines the distributions of sub-communities per community. **f–i**, Probability distributions of damping values $q^{(h)}$. The hierarchy model (**h**) assumes a constant damping value for all levels. Maps of of L_1 communities are not shown as in other countries due to our non-disclosure agreement with the data providers from Country X.

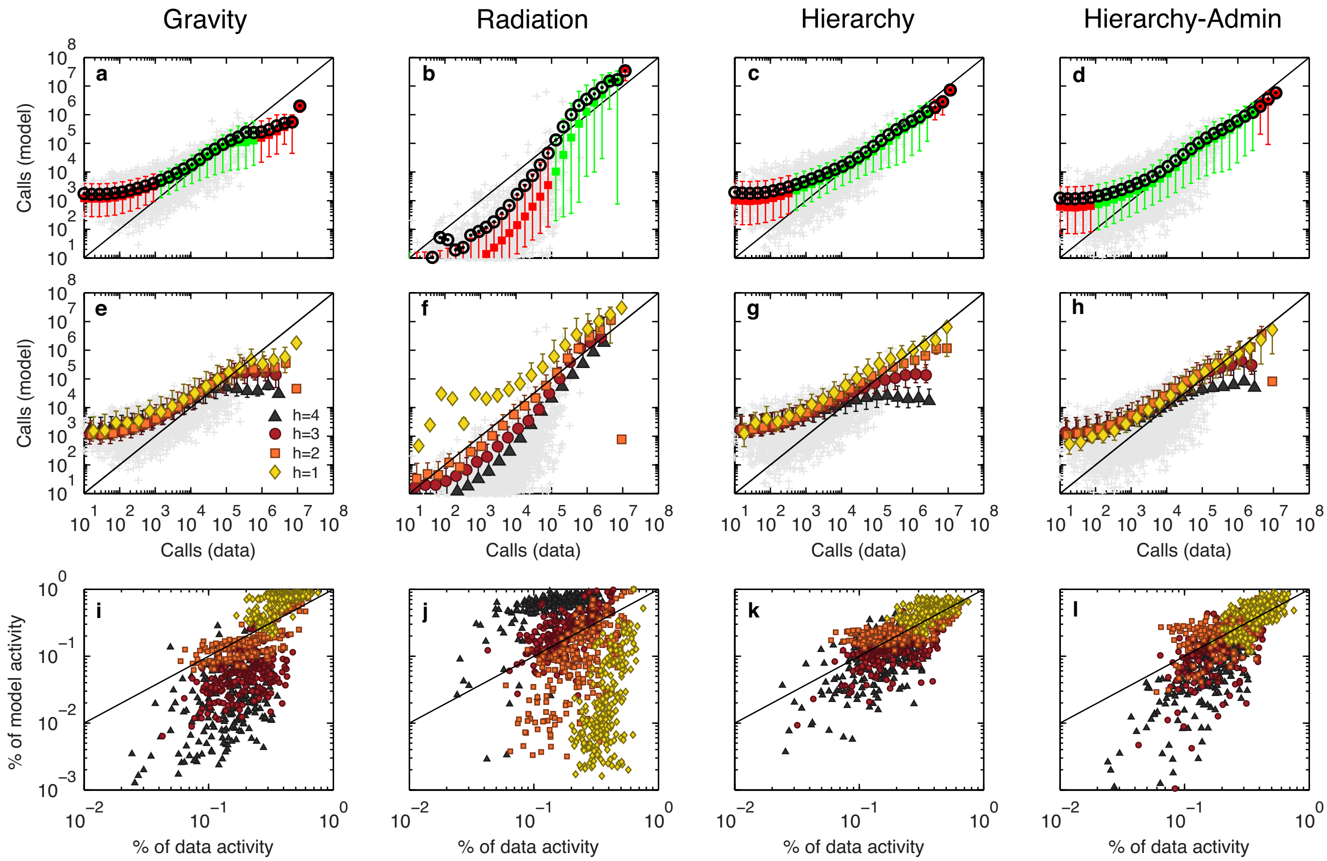

Figure S5. Comparison of model predictions in Portugal. a–d, Comparison of the actual total communication to the predicted communication for each pair of distinct locations, for the (**a**) gravity, (**b**) radiation, (**c**) hierarchy, and (**d**) administrative models. Gray markers are scatter plots for each pair of locations. A box is colored green if the equality line $y = x$ lies between the 9th and 91th percentiles in that bin and is red otherwise. Red boxes hence emphasize significant biases of the models. Black circles correspond to the average total communication of the pairs of locations in that bin. **e–h**, Goodness of prediction with respect to the hierarchical distance h, for the (**e**) gravity, (**f**) radiation, (**g**) hierarchy, and (**h**) administrative models. Gray markers are scatter plots for each pair of locations. Error bars show the corresponding 9th and 91th percentiles of total communication values. **i–l**, For each L3 community, comparison of the fractions of activity of model versus data between that L3 community and L3 communities at different hierarchical distances, for the (**i**) gravity, (**j**) radiation, (**k**) hierarchy and (**l**) administrative models.

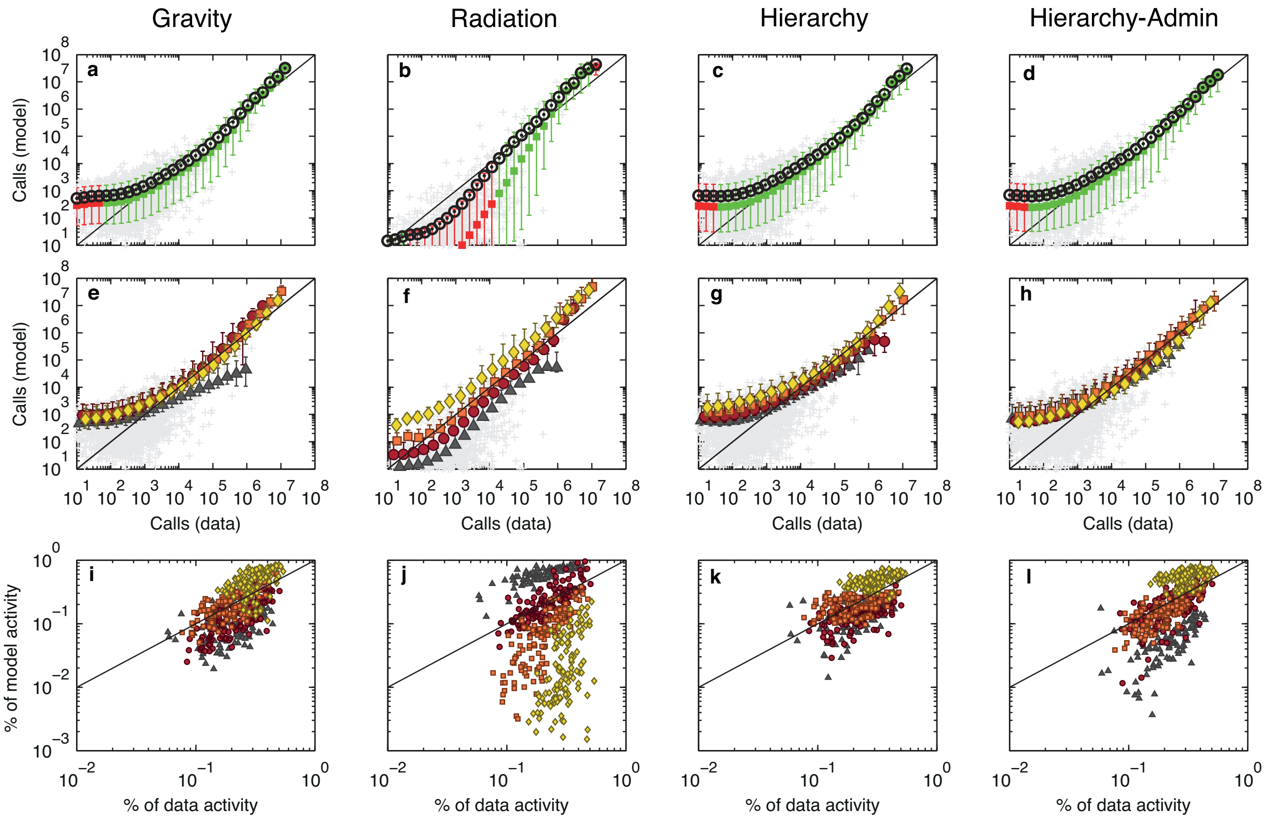

Figure S6. **Comparison of model predictions in France. a–d**, Comparison of the actual total communication to the predicted communication for each pair of distinct locations, for the (**a**) gravity, (**b**) radiation, (**c**) hierarchy, and (**d**) administrative models. Gray markers are scatter plots for each pair of locations. A box is colored green if the equality line $y = x$ lies between the 9th and 91th percentiles in that bin and is red otherwise. Red boxes hence emphasize significant biases of the models. Black circles correspond to the average total communication of the pairs of locations in that bin. **e–h**, Goodness of prediction with respect to the hierarchical distance h, for the (**e**) gravity, (**f**) radiation, (**g**) hierarchy, and (**h**) administrative models. Gray markers are scatter plots for each pair of locations. Error bars show the corresponding 9th and 91th percentiles of total communication values. **i–l**, For each L3 community, comparison of the fractions of activity of model versus data between that L3 community and L3 communities at different hierarchical distances, for the (**i**) gravity, (**j**) radiation, (**k**) hierarchy and (**l**) administrative models.

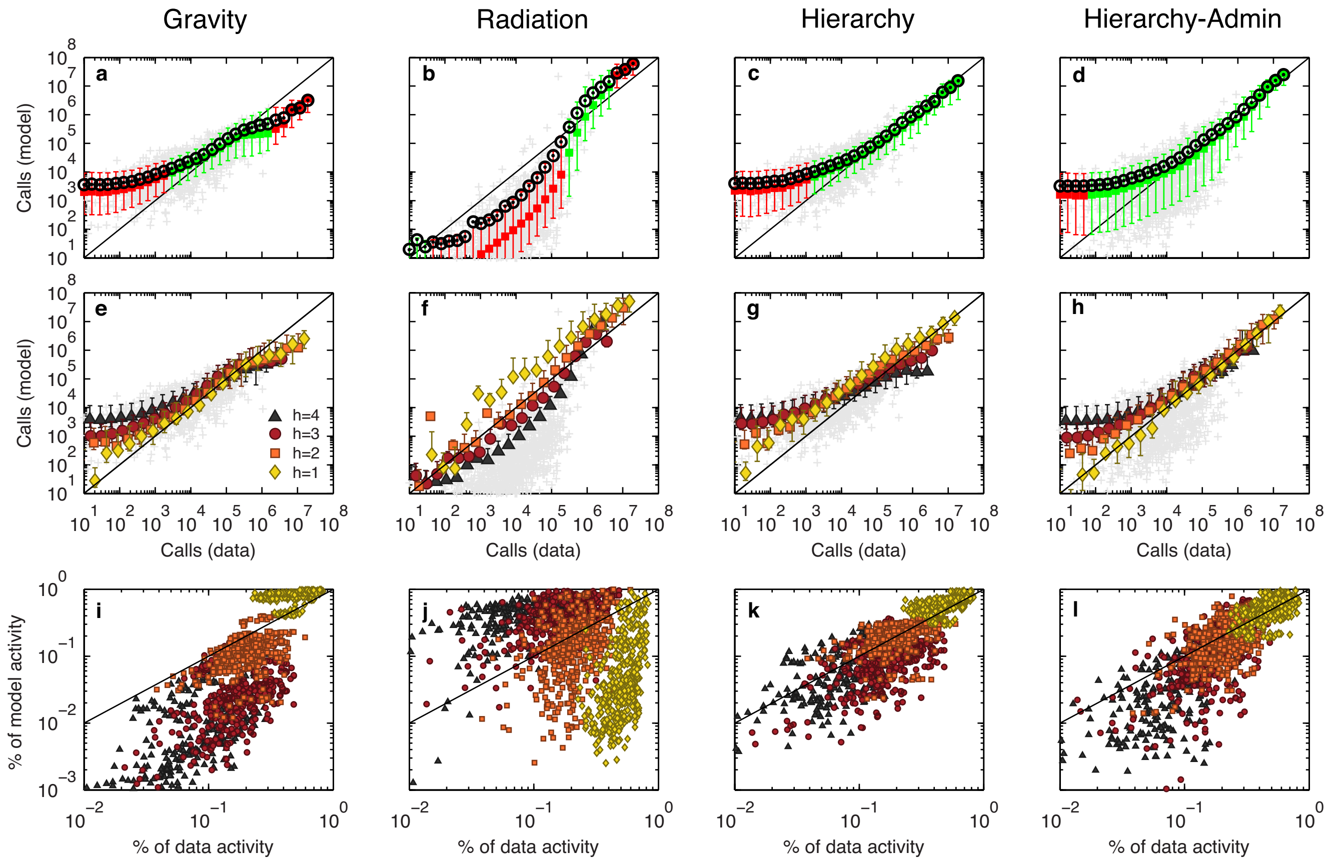

Figure S7. **Comparison of model predictions in Ivory Coast. a–d**, Comparison of the actual total communication to the predicted communication for each pair of distinct locations, for the (**a**) gravity, (**b**) radiation, (**c**) hierarchy, and (**d**) administrative models. Gray markers are scatter plots for each pair of locations. A box is colored green if the equality line $y = x$ lies between the 9th and 91th percentiles in that bin and is red otherwise. Red boxes hence emphasize significant biases of the models. Black circles correspond to the average total communication of the pairs of locations in that bin. **e–h**, Goodness of prediction with respect to the hierarchical distance h, for the (**e**) gravity, (**f**) radiation, (**g**) hierarchy, and (**h**) administrative models. Gray markers are scatter plots for each pair of locations. Error bars show the corresponding 9th and 91th percentiles of total communication values. **i–l**, For each L3 community, comparison of the fractions of activity of model versus data between that L3 community and L3 communities at different hierarchical distances, for the (**i**) gravity, (**j**) radiation, (**k**) hierarchy and (**l**) administrative models.

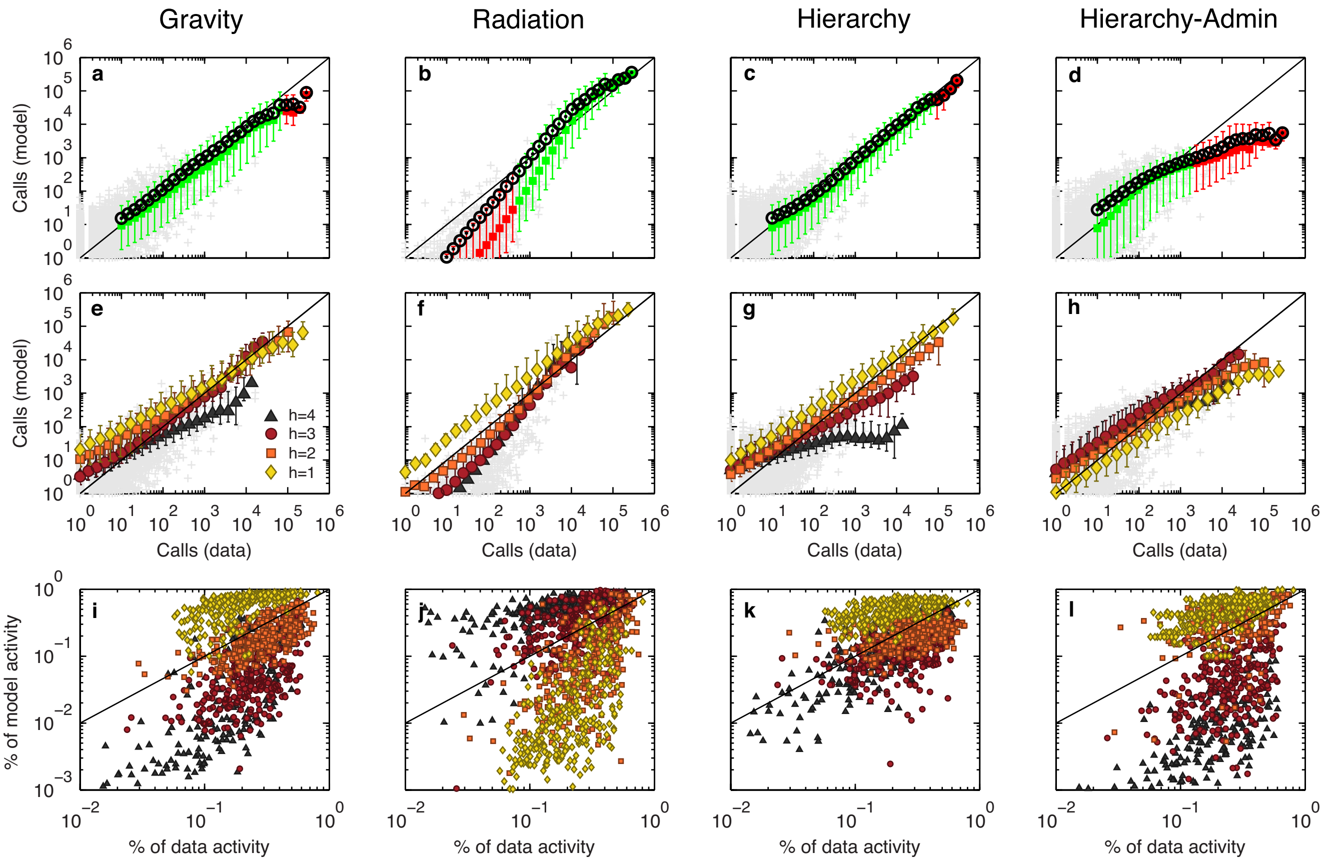

Figure S8. **Comparison of model predictions in Country X. a–d**, Comparison of the actual total communication to the predicted communication for each pair of distinct locations, for the (**a**) gravity, (**b**) radiation, (**c**) hierarchy, and (**d**) administrative models. Gray markers are scatter plots for each pair of locations. A box is colored green if the equality line $y = x$ lies between the 9th and 91th percentiles in that bin and is red otherwise. Red boxes hence emphasize significant biases of the models. Black circles correspond to the average total communication of the pairs of locations in that bin. **e–h**, Goodness of prediction with respect to the hierarchical distance h, for the (**e**) gravity, (**f**) radiation, (**g**) hierarchy, and (**h**) administrative models. Gray markers are scatter plots for each pair of locations. Error bars show the corresponding 9th and 91th percentiles of total communication values. **i–l**, For each L3 community, comparison of the fractions of activity of model versus data between that L3 community and L3 communities at different hierarchical distances, for the (**i**) gravity, (**j**) radiation, (**k**) hierarchy and (**l**) administrative models.

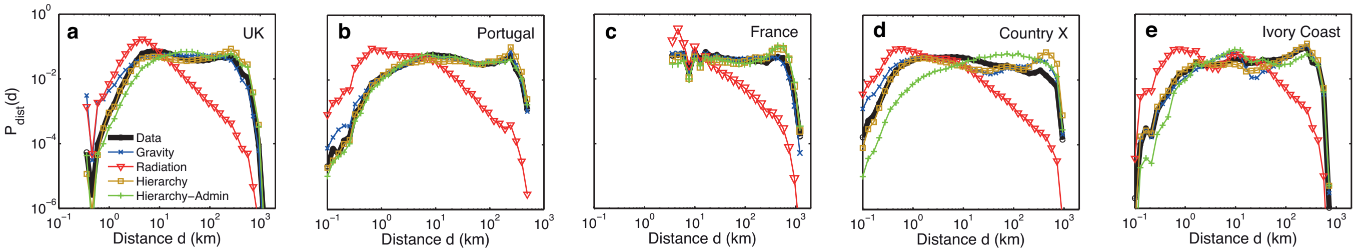

Figure S9. Comparing the model predictions. The proportion $P_{dist}(r)$ of communication occurring between two locations at a distance r (in km) from each other, is measured in the data and in the models. The radiation model is characterized by a lower than actual proportion of communication between distant (more than 100km) locations up to two orders of magnitude. It also presents a higher than actual proportion of communication between close (less than 10km with a peak between 0.5 and 5 km depending on the country) locations up to one order of magnitude. The gravity model presents in all countries a higher than actual proportion of communication between very close locations (100m-1km). In general, it also overestimates the low-range fluxes by 2 to 3 orders of magnitude and slightly underestimates the top-range fluxes. The hierarchy model fits almost perfectly at low distances (less than 10km). Depending on the country, it only deviates slightly from the data at top-range fluxes or estimates them properly. The fit of the hierarchy-admin model depends strongly on the country, but qualitatively comparable to the gravity and hierarchy models.

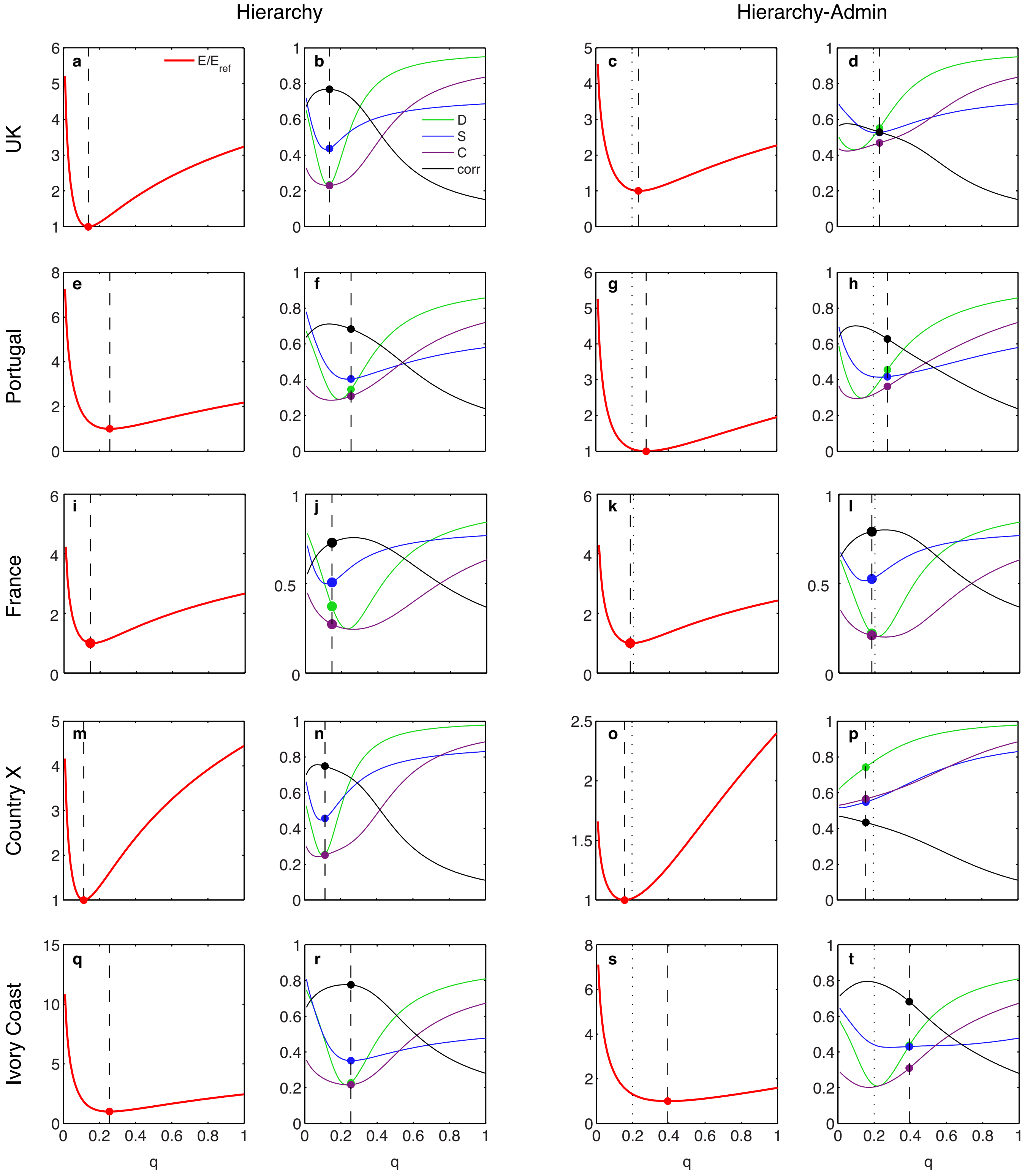

Figure S10. Stability analysis: benchmark measures of the Hierarchy and Hierarchy-Admin models with varying parameter q. The dashed lines and circle markers show the 'optimal' values of q (reported in Supplementary Table 2) minimizing the deviance E. In all countries, either for the hierarchy or hierarchy-admin model, these optimal values of q are also close to those minimizing the Dice (D), Sorensen (S) and Cosine (C) distances and maximizing the correlation ($corr$) between the data and the models. These optimal values are also stable, in the sense that close values of q (roughly between 0.1 and 0.3) still provide benchmark measures close to their optimum. The dotted lines in the case of the Hierarchy-Admin model indicate the damping value $q = 0.2$ matching robustly all countries.

Supplementary Text

Data partition versus administrative regions

Partition overlap measures

We use three classical measures of clustering similarity to quantify partition overlaps, i.e. of how well two different partitions of the same set of locations match: Rand's criterion $\mathscr{R}$[1], Jaccard index $\mathscr{J}$[2] and the Fowlkes and Mallows index $\mathscr{F}$[3]. Consider two partitions $\mathscr{C}$ and $\mathscr{C}'$ of a set of n nodes and note

- n_{11} the number of pairs of nodes in the same community both in $\mathscr{C}$ and $\mathscr{C}'$;
- n_{01} the number of pairs of nodes not in the same community in $\mathscr{C}$ but in the same community in $\mathscr{C}'$;
- n_{10} the number of pairs of nodes in the same community in $\mathscr{C}$ but not in the same community in $\mathscr{C}'$;
- n_{00} the number of pairs of nodes in different communities both in $\mathscr{C}$ and $\mathscr{C}'$.

All types of pairs being taken into account, we have $n_{11}+n_{01}+n_{10}+n_{00}=n(n-1)/2$. The $\mathscr{R}$, $\mathscr{J}$ and $\mathscr{F}$ indices are then defined by:

$$\mathscr{R}=\frac{2(n_{11}+n_{00})}{n(n-1)}, \qquad \mathscr{J}=\frac{n_{11}}{n_{11}+n_{10}+n_{01}}, \qquad \mathscr{F}=\frac{n_{11}}{\sqrt{(n_{11}+n_{10})(n_{11}+n_{01})}} \tag{1}$$

which are different ways of quantifying how well the partitions match pairs of nodes. The values of $\mathscr{R}, \mathscr{J}, \mathscr{F}$ lie between 0 and 1, and values close to 1 indicate a perfect match between the two compared partitions. However, even for the case of two completely unrelated clusterings, all indices are in general strictly larger than zero, more so for $\mathscr{R}$[3]. Therefore, to have a baseline, we calculated the average indices over 10000 random shufflings of the nodes's clusters, denoted by $\bar{\mathscr{R}}_{\mathrm{r}}$, $\bar{\mathscr{J}}_{\mathrm{r}}$ and $\bar{\mathscr{F}}_{\mathrm{r}}$ (see Table S4).

Partition overlap results

The overlap measures between L_1, L_2 and L_3 level partitions and the corresponding administrative divisions of the considered countries are given in Table S4, together with p-values assessing the statistical significance of the partition with respect to a null model in which communities of cell towers were randomly reshuffled, quantitatively confirm the similarity between the administrative partitions (we choose to refer to the european *Nomenclature of Territorial Units for Statistics* - or NUTS - standard for the european countries) and the Combo partition. For example, the L_1 UK partitioning shows values of $\mathscr{R}=0.954$ with a baseline $\bar{\mathscr{R}}_{\mathrm{r}}=0.825$, $\mathscr{J}=0.618$ with a baseline $\bar{\mathscr{J}}_{\mathrm{r}}=0.049$, and $\mathscr{F}=0.769$ with a baseline $\bar{\mathscr{F}}_{\mathrm{r}}=0.096$ - with all significance measures ($p<10^{-4}$) indicating a good match between the administrative and the Combo partitions. Going a step further and comparing the L_2 and L_3 communities with NUTS regions of corresponding levels, the match between the Combo and administrative partitions is still good, as indicated by significantly higher than average overlap measures. The L_2 UK partitioning hence shows values of $\mathscr{R}=0.972$ with a baseline $\bar{\mathscr{R}}_{\mathrm{r}}=0.957$, $\mathscr{J}=0.222$ with a baseline $\bar{\mathscr{J}}_{\mathrm{r}}=0.007$, and $\mathscr{F}=0.439$ with a baseline $\bar{\mathscr{F}}_{\mathrm{r}}=0.018$ and the L_3 UK partitioning shows values of $\mathscr{R}=0.988$ with a baseline $\bar{\mathscr{R}}_{\mathrm{r}}=0.986$, $\mathscr{J}=0.099$ with a baseline $\bar{\mathscr{J}}_{\mathrm{r}}=0.001$, and $\mathscr{F}=0.292$ with a baseline $\bar{\mathscr{F}}_{\mathrm{r}}=0.004$. Note that while all these values stay significant, the differences between the Combo / administrative overlap values and the random / administrative overlap values decrease when we look at more fine-grained partitions. This has to be expected since the deviations between the Combo and administrative partition can only increase with the level of partition as the deviations at a given level automatically impact the successive levels. Similar results can be drawn from the other countries, see Table S4.

Table S4 also display the modularity scores Q^*_{Combo} and Q^*_{off} of the Combo and administrative partitions at the different levels. To be consistent with the procedure of the Combo algorithm, which builds the level $n+1$ subpartition by decomposing each community of the level n partition, these modularity scores indicate in case of L_2 (resp. L_3) partitions the average modularity score of each subpartition defined with respect to a subnetwork inside each corresponding L_1 (resp. L_2) community. Our measures show that the Combo partition always has a better modularity score than the administrative partition. The modularity scores also decrease when we look at higher level partitions, indicating that the L_1 communities are the most relevant.

Interpretation

The similarity between the regions emerging from the communication network through the Combo procedure and the administrative boundaries can be interpreted as a natural evidence towards the latter's validity[4,5]. In view of the deviation between the two partitions, Combo partitioning appears to be more aligned with human interactions, as measured by the modularity score, suggesting that the border of administrative regions sometimes deviates from the underlying reality of

interactions. Most interestingly, the partitioning created by our approach provides a unified hierarchical framework to compare the geographical structure of human interactions in different countries, which present an important alternative to the administrative boundaries whose shape and number depend substantially on the historical and political context of each country as well as particular local regional delineation policy.

Table S4. Overlap between the administrative regions and the community found by the Combo algorithm.

Country	Network	N_{off}	N_{combo}	Q^*_{off}	Q^*_{Combo}	$\mathscr{R}(\bar{\mathscr{R}}_{\rm r})$	$\mathscr{J}(\bar{\mathscr{J}}_{\rm r})$	$\mathscr{F}(\bar{\mathscr{F}}_{\rm r})$
UK	NUTS1/L_1	11	16	0.642	0.657	0.954(0.825)	0.618(0.049)	0.769(0.096)
UK	NUTS2/L_2	36	150	0.490	0.631	0.972(0.957)	0.222(0.007)	0.439(0.018)
UK	NUTS3/L_3	133	917	0.415	0.472	0.988(0.986)	0.099(0.001)	0.292(0.004)
Portugal	NUTS2/L_1	5	7	0.445	0.491	0.859(0.669)	0.496(0.113)	0.671(0.206)
Portugal	NUTS3/L_2	28	44	0.358	0.478	0.935(0.881)	0.314(0.027)	0.523(0.057)
Portugal	NUTS4/L_3	275	226	0.359	0.361	0.984(0.975)	0.230(0.005)	0.411(0.011)
France	NUTS1/L_1	22	14	0.638	0.645	0.964(0.874)	0.579(0.033)	0.748(0.066)
France	NUTS2/L_2	96	108	0.486	0.592	0.994(0.978)	0.609(0.006)	0.758(0.011)
France	NUTS3/L_3	335	609	0.347	0.405	0.997(0.994)	0.336(0.001)	0.522(0.003)
Country X	NUTS1/L_1	-	11	0.420	0.453	0.862(0.756)	0.342(0.073)	0.518(0.139)
Country X	NUTS2/L_2	-	81	0.312	0.480	0.874(0.843)	0.127(0.018)	0.338(0.054)
Country X	NUTS3/L_3	-	388	0.315	0.341	0.907(0.901)	0.031(0.003)	0.160(0.018)
Ivory Coast	regions/L_1	19	19	0.666	0.807	0.845(0.761)	0.264(0.055)	0.474(0.118)
Ivory Coast	departments/L_2	50	209	0.306	0.589	0.900(0.881)	0.097(0.010)	0.308(0.035)
Ivory Coast	prefectures/L_3	255	1401	0.277	0.452	0.966(0.962)	0.057(0.002)	0.236(0.009)

The "Network" column indicates the levels of the administrative partition and of the community partition that are compared[5]. Columns N_{off} and N_{combo} respectively refer to the number of NUTS regions and the number of communities found by the community detection algorithm at the considered level. Columns Q^*_{off} and Q^*_{Combo} indicate the average modularity score of all the administrative or Combo sub-partitions at the considered level. $\bar{\mathscr{R}}_{\rm r}$, $\bar{\mathscr{J}}_{\rm r}$ and $\bar{\mathscr{F}}_{\rm r}$ give the baselines for Rand's criterion $\mathscr{R}$, the Jaccard index $\mathscr{J}$ and the Fowlkes-Mallows index $\mathscr{F}$. The closer $\mathscr{R}$, $\mathscr{J}$ or $\mathscr{F}$ to 1, the better the overlap of the detected communities with the administrative regions. The baseline values of the similarity indices are computed on 10000 random shuffling of the nodes's clusters. For the 3 used measures, none of these random shufflings is more similar to the administrative partition than the partition found by the community detection algorithm: the statistical significances of all the similarities have a p-value $< 10^{-4}$.

Central Place Theory

Classical theories of urban planning have traditionally suggested economical and geographic laws to systematically determine the arrangement of towns and cities. In particular, the central place theory (CPT) developed by Christaller[7,8] seeks to explain the number, size and location of human settlements in an urban system. The basic assumption of the theory is that settlements function as 'central places' providing services to surrounding areas. The hierarchy of the cities is based on the range of goods and services they provide. Low order goods and services (groceries, bakeries, post offices) are present in all places, including small and large centers. Higher order goods and services (jewellery, large malls, universities) are only present in large centers, which are less numerous. These centers are supported by a large population, including its own and those of the surrounding smaller centers. The lowest settlements should form an hexagonal lattice, this being the most efficient regular pattern to serve areas without any overlap (in terms of radial distance and perimeter for a fixed area). Settlements of higher order (villages, cities) should then be regularly spaced on an hexagonal pattern of higher radius, with their centers placed on centers of hexagons of the lowest order.

Christaller defined K-hexagonal landscapes as arrangements where each higher order settlement is supported by $K-1$ lower order settlements and itself. Christaller and later Lösch[9] both developed arguments over which value of K is adapted to describe different situations. For example, $K=3$ is suited for sharing local goods (marketing principle), $K=4$ is suited for reducing cost of transport (traffic principle) while $K=7$ - a case where each satellite depends only on one center - is suited for political stability (administrative principle). Christaller conceived these models as hierarchical, with all higher order places in the hexagon surrounded by lower order places to explain not only local but regional economics and spatialization of urban centers, the value of K possibly changing from level to level.

Filling the gap between CPT and reality, several distortions to the original model have been introduced over time to account for inhomogeneous population densities, resource locations, or specialization of cities. However, nowadays CPT is not a part of modern regional science and it has been criticized for being a static theory, not explaining how central places emerge and

develop[10]. It has also been shown that while CPT is ideally suited to describe agricultural areas, it is less relevant to describe industrial areas which are highly diversified in nature. But despite its imperfections, CPT still remains one of the strongest economic theories for understanding the spatial organization of the society as the hierarchy of urban centers. It has been applied in regional and urban economies, describing the location of trade and service activity, and for describing consumer market-oriented manufacturing.

Models' variations

In parallel with the models presented in the main text, we also tested different variations of the gravity, radiation and hierarchy models to predict human interactivity. In the following section, we present the definition of these variations and standard benchmark measures quantifying how well these models fit the data. As for the models presented in the main text, we always use the total amount of communication w_i originating from a location i as a proxy for its population.

Definitions

Radiation model versions The radiation model is a parameter-free model recently introduced in the context of migration patterns[11]. Given the distance d_{ij} between two locations i and j, the model predicts that the flux of individual moves T_{ij} between those two locations will depend on the population at the origin, the population at the destination and on the population s_{ij} within the circle of radius d_{ij} centered on the origin location i. Applied to our case, the radiation model is written as

$$T_{ij}^{Rad} = C_i \frac{w_i w_j}{(w_i + s_{ij})(w_i + w_j + s_{ij})} = C_i w_i \left(\frac{1}{w_i + s_{ij}} - \frac{1}{w_i + w_j + s_{ij}} \right), \tag{2}$$

where $s_{ij} = \sum_{k, 0<d_{ik}<d_{ij}} w_k$ is the total amount of communication originating from locations at a distance shorter than d_{ij} from location i and C_i is a normalization factor ensuring that the predicted total activity of each node is the same as the actual one, i.e. $\sum_{j \neq i} T_{ij}^{Rad} = \sum_{j \neq i} T_{ij}$. The model is otherwise parameter-free.

We also applied the generalized version of the radiation model proposed in[12], introducing a parameter λ which can be interpreted in our case as a fraction of individuals people will not consider as potential contacts because of lack of information about them of other reasons. This version of the radiation model can be written as

$$T_{ij}^{GenRad} = C_i w_i \frac{1}{1-\lambda} \left(\frac{1-\lambda^{w_i+s_{ij}}}{w_i + s_{ij}} - \frac{1-\lambda^{w_i+w_j+s_{ij}}}{w_i + w_j + s_{ij}} \right). \tag{3}$$

where C_i is again a normalization factor ensuring that $\sum_{j \neq i} T_{ij}^{GenRad} = \sum_{j \neq i} T_{ij}$. Notice that the case $\lambda = 0$ corresponds to the original radiation model.

Gravity models The gravity model is one of the oldest models describing human mobility and interaction, formulated in analogy to Newton's law of gravity. Here interaction strength or mobility fluxes between a source i and a destination j are originally proposed to be related to a function of the distance d_{ij} between the two locations and the product of the (powers of) population at the source an at the destination, $T_{ij}^{Grav} \sim w_i^{\alpha} w_j^{\beta} f(d_{ij})$. We tested the two classical forms of the gravity model using a power or an exponential law as deterrence function:

$$T_{ij}^{GravPL} = C w_i^{\alpha} w_j^{\beta} d_{ij}^{\gamma}, \tag{4}$$

$$T_{ij}^{GravEXP} = C w_i^{\alpha} w_j^{\beta} \exp(-d_{ij}/d_0) \tag{5}$$

where in both cases C is a global normalization constant ensuring that $\sum_{i, j \neq i} T_{ij}^{Grav} = \sum_{i, j \neq i} T_{ij}$ and α, β, γ, d_0 are parameters to fit. For example, taking the logarithm on both sides of Eq. (4), we obtain $\log T_{ij}^{GravPL} = \log C + \alpha \log w_i + \beta \log w_j + \gamma \log d_{ij}$. We then use, as is customary, $\log T_{ij}^{GravPL}$ to estimate the different parameters through a regression analysis[13]. We selected the set of parameters that minimized the deviance E (see definition in main text, Methods section). We also computed versions of these models where the population exponents are fixed, i.e. $\alpha = \beta = 1$.

At this point, we observe an often overseen difference between the gravity model relying on a single global normalization factor C and the radiation model relying on local normalization factors $\{C_i\}$ - one for each location. One could argue that this difference gives an advantage to the radiation model. For the sake of comparison, we then also tested *locally constrained* gravity models that can be written as:

$$T_{ij}^{GravPLloc} = C_i w_i w_j d_{ij}^{\gamma} \tag{6}$$

$$T_{ij}^{GravEXPloc} = C_i w_i w_j \exp(-d_{ij}/d_0) \tag{7}$$

where in each case C_i is a normalization factor ensuring that the predicted total activity of each node is the same as the actual one. This type of constrained model was already presented in[14] and more recently in[15].

Hierarchical models The general idea behind the hierarchical model is to simply replace the actual distance used in the gravity models by a hierarchical distance. In its most generic definition, the hierarchical model predicts an interaction strength between location i and j to be written as $T_{ij}^{Hier} = C_i w_i^{\alpha} w_j^{\beta} f(h_{ij})$, where h_{ij} is the hierarchical distance between these two locations based on the Combo partition or any other partition and f is a deterrence function. As it is done for the gravity model, one could a priori choose any deterrence function. We tested different simple forms of hierarchy models, using the hierarchical distances $\{h_{ij}\}$ provided either by the Combo partition or the administrative partition. Hierarchy models using a global normalization framework - as the usual gravity models do - read:

$$T_{ij}^{HierEXP} = C w_i^{\alpha} w_j^{\beta} q^{h_{ij}} \tag{8}$$
$$T_{ij}^{HierPL} = C w_i^{\alpha} w_j^{\beta} h_{ij}^{\gamma}, \tag{9}$$

where C is a global normalization constant, and α, β, γ and q are parameters which we fit by minimizing the deviance E, see below. We also computed versions of these models where the population exponents are fixed, i.e. $\alpha = \beta = 1$.

The locally constrained versions of models given in Eqs. (8) and (9) with fixed population exponent read:

$$T_{ij}^{HierEXPloc} = C_i w_i w_j q^{h_{ij}} \tag{10}$$
$$T_{ij}^{HierPLloc} = C_i w_i w_j h_{ij}^{\gamma}, \tag{11}$$

It turns out that the model given by Eq (10) is naturally related to the notion of damping parameter. Indeed, assuming $\beta = 1$ in the generic functional form $T_{ij}^{Hier} = C_i w_i^{\alpha} w_j^{\beta} f(h_{ij})$ and taking into account that the normalization factor C_i ensures $w_i^{Hier} = \sum_j T_{ij}^{Hier(h)} = w_i$ implies

$$\begin{aligned} T_i^{Hier(h)} &= \sum_{j,h_{ij}=h} T_{ij}^{Hier} \\ &= C_i w_i^{\alpha} f(h) \sum_{j,h_{ij}=h} w_j \\ &= C_i w_i^{\alpha} f(h) \sum_{j,h_{ij}=h} w_j^{Hier(h)} \\ &= C_i w_i^{\alpha} f(h) W_i^{Hier(h)} \end{aligned}$$

and thus

$$q_i^{Hier(h)} = \frac{T_i^{Hier(h+1)}}{T_i^{Hier(h)}} \frac{W_i^{Hier(h)}}{W_i^{Hier(h+1)}} = \frac{f(h+1)}{f(h)}, \tag{12}$$

an equation which immediately implies that choosing an exponential deterrence function $f(h) = q^h$ will ensure a constant damping parameter with respect to the locations and hierarchy levels $q_i^{Hier(h)} = q$ for all i,h.

Hierarchical-Admin models The hierarchical models rely on the notion of hierarchical distances between locations, which depend on a given partition. For cases when the communication network or it's partitioning are not known, we can as well defined a model based on Administrative partitions of the countries. In the following, we refer to hierarchy models based on Administrative partition as 'hierarchy-admin' models.

Analysis

Benchmark measures (as defined in main text) of the different models along with their fitted parameters are reported in Tables S5 and S6. We make a number of remarkable observations:

- In every country and according to all benchmark measures, the generalized radiation model is significantly more appropriate than the original one to describe our communication networks (e.g. in UK, the cosine distance to the data goes from 0.344 in the original radiation model to 0.195 in the generalized radiation model, similarly the correlation to the data goes from 0.656 to 0.805).
- Locally constrained models always perform better compared to their 'global normalization' counterpart.
- When using an exponential deterrence function, the hierarchy models based on our Combo partition are always significantly better than the corresponding gravity models, with respect to the deviance or any other benchmark measure. E.g. in UK, the cosine distance goes from 0.595 in the 'gravity EXP' model to 0.278 in the 'Hierarchy EXP' model, similarly the correlation to the data goes from 0.402 to 0.72. The Hierarchy models using the administrative partition are also slightly better than the corresponding gravity models, except in Country X where we already observed some bias due to the administrative borders (see section II-D above).
- When using a power law deterrence function, the best model can vary with respect to the benchmark measure and the studied country. The 'Gravity PL loc' model is in general the best one.
- As one could expect, the models where the population exponent α and β are not constrained always have a lower deviance E than the corresponding models with $\alpha = \beta = 1$ (but be aware that a correct comparison of the performance of the models based on the deviance should also take the number of parameter into account). According to the other benchmark measures (not used for the fitting), the unconstrained models are also most often better fit than the constrained ones (e.g. their correlation to data is higher 12 times out of 18) and when it's not the case the difference between the fit measures is small.
- In general, the gravity PL model is better than the gravity EXP model. It is the best one regarding all benchmark measures in UK and Country X, and two out of five measures in Portugal.
- The hierarchy EXP models always fit better than the hierarchy PL models, all measures and countries considered.

The 20 tested models can be ranked according to the average of the benchmark measures taken over all countries, see Table S7. While on average the best model is always the constrained 'Gravity PL loc' model, the 'hierarchy EXP loc' model presented in the main text is a close second (and first in some countries). 7 of the top 10 models are versions of the hierarchy model. In particular, the 'hierarchy-admin EXP loc' (presented in main text), the 'hierarchy PL loc' and 'hierarchy PL' based on a power law deterrence function also outperform the state-of-the-art radiation and gravity PL models.

Table S5. Benchmark goodness fit measures The Dice (D), Sorensen (S), Cosine (C) and deviance (E) are four different fit values measuring a distance between the actual and modeled networks. The correlation *corr* measures a similarity between the model and the data. The different parameters of the gravity and hierarchy models were chosen to minimize the value of E. Stars (*) denote models where we imposed the population exponents α and β to be equal to 1. The rows corresponding to models presented in the main text are highlighted.

Country / Model	$E*10^{-9}$	D	S	C	corr	params
UK / radiation	1622.9	0.624	0.632	0.344	0.656	
UK / gen. radiation	818.3	0.236	0.444	0.195	0.805	$1-\lambda = 8.65\times10^{-10}$
UK / gravity EXP	941.1	0.777	0.579	0.595	0.402	$\alpha = 0.78$, $\beta = 0.78$, $d_0 = 63.8km$
UK / hierarchy EXP	547.7	0.278	0.447	0.278	0.720	$\alpha = 0.90$, $\beta = 0.90$, $q = 0.138$
UK / hierarchy-admin EXP	741.2	0.580	0.536	0.512	0.485	$\alpha = 0.90$, $\beta = 0.90$, $q = 0.374$
UK / gravity EXP*	969.4	0.749	0.583	0.612	0.384	$d_0 = 67.3km$
UK / hierarchy EXP*	553.8	0.291	0.448	0.288	0.711	$q = 0.139$
UK / hierarchy-admin EXP*	747.7	0.566	0.534	0.520	0.477	$q = 0.235$
UK / gravity EXP loc	839.6	0.726	0.555	0.547	0.450	$d_0 = 56.1km$
UK / hierarchy EXP loc	464.9	0.233	0.437	0.231	0.768	$q = 0.139$
UK / hierarchy-admin EXP loc	662.5	0.558	0.527	0.470	0.239	$q = 0.239$
UK / gravity PL	494.7	0.456	0.448	0.456	0.543	$\alpha = 0.65$, $\beta = 0.65$, $\gamma = -1.46$
UK / hierarchy PL	649.9	0.334	0.486	0.326	0.673	$\alpha = 0.91$, $\beta = 0.91$, $\gamma = -4.23$
UK / hierarchy-admin PL	768.7	0.571	0.542	0.519	0.478	$\alpha = 0.90$, $\beta = 0.90$, $\gamma = -3.10$
UK / gravity PL*	562.8	0.433	0.467	0.431	0.567	$\gamma = -1.36$
UK / hierarchy PL*	655.2	0.354	0.488	0.334	0.665	$\gamma = -4.21$
UK / hierarchy-admin PL*	774.8	0.560	0.541	0.527	0.471	$\gamma = -3.09$
UK / gravity PL loc	351.5	0.218	0.375	0.216	0.783	$\gamma = -1.57$
UK / hierarchy PL loc	555.6	0.290	0.481	0.288	0.711	$\gamma = -4.22$
UK / hierarchy-admin PL loc	679.9	0.540	0.533	0.468	0.529	$\gamma = -3.08$
Portugal / radiation	314.1	0.781	0.739	0.476	0.525	
Portugal / gen. radiation	78.4	0.472	0.420	0.423	0.563	$1-\lambda = 4.22\times10^{-10}$
Portugal / gravity EXP	96.20	0.718	0.461	0.577	0.400	$\alpha = 0.87$, $\beta = 0.86$, $d_0 = 92.9km$
Portugal / hierarchy EXP	71.86	0.465	0.420	0.422	0.564	$\alpha = 0.91$, $\beta = 0.89$, $q = 0.244$
Portugal / hierarchy-admin EXP	83.98	0.605	0.441	0.501	0.480	$\alpha = 0.83$, $\beta = 0.82$, $q = 0.343$
Portugal / gravity EXP*	97.15	0.700	0.460	0.577	0.399	$d_0 = 95.6km$
Portugal / hierarchy EXP*	72.45	0.455	0.419	0.426	0.561	$q = 0.281$
Portugal / hierarchy-admin EXP*	85.65	0.580	0.441	0.500	0.482	$q = 0.352$
Portugal / gravity EXP loc	92.08	0.684	0.455	0.546	0.433	$d_0 = 82.0km$
Portugal / hierarchy EXP loc	66.66	0.346	0.404	0.308	0.683	$q = 0.258$
Portugal / hierarchy-admin EXP loc	74.20	0.456	0.416	0.362	0.627	$q = 0.278$
Portugal / gravity PL	79.80	0.865	0.419	0.844	0.145	$\alpha = 0.81$, $\beta = 0.79$, $\gamma = -0.71$
Portugal / hierarchy PL	77.79	0.473	0.443	0.450	0.536	$\alpha = 0.91$, $\beta = 0.90$, $\gamma = -2.76$
Portugal / hierarchy-admin PL	86.87	0.593	0.452	0.511	0.470	$\alpha = 0.85$, $\beta = 0.83$, $\gamma = -2.28$
Portugal / gravity PL*	81.88	0.793	0.420	0.781	0.205	$\gamma = -0.69$
Portugal / hierarchy PL*	78.32	0.468	0.441	0.454	0.532	$\gamma = -2.73$
Portugal / hierarchy-admin PL*	88.23	0.572	0.450	0.509	0.473	$\gamma = -2.24$
Portugal / gravity PL loc	66.37	0.403	0.386	0.386	0.602	$\gamma = -0.92$
Portugal / hierarchy PL loc	73.29	0.373	0.433	0.352	0.637	$\gamma = -2.89$
Portugal / hierarchy-admin PL loc	77.05	0.429	0.431	0.363	0.625	$\gamma = -2.72$
France / radiation	227.758	0.618	0.647	0.270	0.730	
France / gen. radiation	109.123	0.426	0.494	0.244	0.756	$1-\lambda = 4.40\times10^{-9}$
France / gravity EXP	139.132	0.453	0.639	0.367	0.632	$\alpha = 0.87$, $\beta = 0.86$, $d_0 = 92.0km$
France / hierarchy EXP	92.284	0.443	0.518	0.330	0.670	$\alpha = 0.89$, $\beta = 0.89$, $q = 0.142$
France / hierarchy-admin EXP	101.248	0.270	0.544	0.266	0.734	$\alpha = 0.90$, $\beta = 0.90$, $q = 0.179$
France / gravity EXP*	142.829	0.370	0.637	0.370	0.630	$d_0 = 98.4km$
France / hierarchy EXP*	94.876	0.616	0.519	0.368	0.632	$q = 0.146$
France / hierarchy-admin EXP*	103.187	0.328	0.539	0.263	0.737	$q = 0.183$
France / gravity EXP loc	116.792	0.378	0.611	0.276	0.724	$d_0 = 96.7km$
France / hierarchy EXP loc	73.524	0.341	0.514	0.267	0.733	$q = 0.158$
France / hierarchy-admin EXP loc	80.686	0.212	0.529	0.207	0.793	$q = 0.192$
France / gravity PL	90.905	0.267	0.524	0.185	0.815	$\alpha = 0.69$, $\beta = 0.69$, $\gamma = -1.44$
France / hierarchy PL	103.181	0.572	0.560	0.427	0.573	$\alpha = 0.91$, $\beta = 0.91$, $\gamma = -4.13$
France / hierarchy-admin PL	110.282	0.329	0.575	0.323	0.677	$\alpha = 0.91$, $\beta = 0.91$, $\gamma = -3.63$
France / gravity PL*	111.425	0.663	0.590	0.313	0.687	$\gamma = -1.16$
France / hierarchy PL*	105.061	0.702	0.558	0.462	0.538	$\gamma = -4.08$
France / hierarchy-admin PL*	111.902	0.377	0.569	0.321	0.679	$\gamma = -3.59$
France / gravity PL loc	71.101	0.237	0.495	0.123	0.877	$\gamma = -1.36$
France / hierarchy PL loc	81.804	0.485	0.559	0.378	0.622	$\gamma = -3.94$
France / hierarchy-admin PL loc	87.132	0.248	0.559	0.246	0.679	$\gamma = -3.52$

Table S6. Benchmark goodness fit measures The Dice (D), Sorensen (S), Cosine (C) and deviance (E) are four different fit values measuring a distance between the actual and modeled networks. The correlation *corr* measures a similarity between the model and the data. The different parameters of the gravity and hierarchy models were chosen to minimize the value of E. Stars (*) denote models where we imposed the population exponents α and β to be equal to 1.

Country / Model	$E*10^{-9}$	D	S	C	corr	params
Country X / radiation	3.701	0.577	0.638	0.356	0.644	
Country X / gen. radiation	1.396	0.244	0.415	0.241	0.759	$1-\lambda = 4.49\times10^{-7}$
Country X / gravity EXP	2.167	0.760	0.531	0.558	0.444	$\alpha = 0.83$, $\beta = 0.80$, $d_0 = 53.2km$
Country X / hierarchy EXP	1.508	0.361	0.483	0.348	0.651	$\alpha = 0.90$, $\beta = 0.87$, $q = 0.117$
Country X / hierarchy-admin EXP	2.439	0.817	0.585	0.645	0.354	$\alpha = 0.82$, $\beta = 0.79$, $q = 0.156$
Country X / gravity EXP*	2.333	0.698	0.533	0.555	0.442	$d_0 = 54.3km$
Country X / hierarchy EXP*	1.534	0.340	0.483	0.340	0.660	$q = 0.118$
Country X / hierarchy-admin EXP*	2.512	0.765	0.587	0.642	0.356	$q = 0.159$
Country X / gravity EXP loc	1.915	0.665	0.488	0.478	0.522	$d_0 = 50.2km$
Country X / hierarchy EXP loc	1.120	0.255	0.456	0.252	0.748	$q = 0.114$
Country X / hierarchy-admin EXP loc	2.076	0.743	0.547	0.565	0.434	$q = 0.158$
Country X / gravity PL	1.483	0.472	0.467	0.470	0.529	$\alpha = 0.81$, $\beta = 0.78$, $\gamma = -1.06$
Country X / hierarchy PL	2.021	0.389	0.565	0.384	0.615	$\alpha = 0.91$, $\beta = 0.88$, $\gamma = -4.43$
Country X / hierarchy-admin PL	2.257	0.794	0.567	0.638	0.361	$\alpha = 0.83$, $\beta = 0.80$, $\gamma = -3.71$
Country X / gravity PL*	1.573	0.409	0.469	0.405	0.595	$\gamma = -1.07$
Country X / hierarchy PL*	2.042	0.378	0.566	0.378	0.622	$\gamma = -4.41$
Country X / hierarchy-admin PL*	2.322	0.742	0.567	0.636	0.363	$\gamma = -3.67$
Country X / gravity PL loc	0.940	0.202	0.382	0.201	0.798	$\gamma = -1.22$
Country X / hierarchy PL loc	1.589	0.310	0.556	0.308	0.692	$\gamma = -4.43$
Country X / hierarchy-admin PL loc	1.882	0.707	0.523	0.545	0.454	$\gamma = -3.77$
Ivory Coast / radiation	268.18	0.701	0.703	0.358	0.645	
Ivory Coast / gen. radiation	72.08	0.373	0.442	0.349	0.638	$1-\lambda = 4.20\times10^{-10}$
Ivory Coast / gravity EXP	74.02	0.680	0.430	0.539	0.434	$\alpha = 0.96$, $\beta = 0.96$, $d_0 = 149.6km$
Ivory Coast / hierarchy EXP	45.85	0.297	0.366	0.374	0.716	$\alpha = 0.93$, $\beta = 0.93$, $q = 0.271$
Ivory Coast / hierarchy-admin EXP	78.71	0.685	0.448	0.553	0.418	$\alpha = 0.96$, $\beta = 0.96$, $q = 0.567$
Ivory Coast / gravity EXP*	74.08	0.673	0.429	0.537	0.436	$d_0 = 149.7km$
Ivory Coast / hierarchy EXP*	46.15	0.285	0.364	0.270	0.720	$q = 0.273$
Ivory Coast / hierarchy-admin EXP*	78.78	0.678	0.447	0.550	0.421	$q = 0.568$
Ivory Coast / gravity EXP loc	65.51	0.567	0.414	0.423	0.561	$d_0 = 112.9km$
Ivory Coast / hierarchy EXP loc	42.90	0.228	0.351	0.217	0.775	$q = 0.255$
Ivory Coast / hierarchy-admin EXP loc	65.98	0.437	0.430	0.309	0.681	$q = 0.394$
Ivory Coast / gravity PL	68.17	0.577	0.413	0.460	0.519	$\alpha = 0.94$, $\beta = 0.94$, $\gamma = -0.51$
Ivory Coast / hierarchy PL	50.89	0.321	0.378	0.317	0.672	$\alpha = 0.93$, $\beta = 0.93$, $\gamma = -2.89$
Ivory Coast / hierarchy-admin PL	80.72	0.692	0.450	0.558	0.412	$\alpha = 0.96$, $\beta = 0.96$, $\gamma = -1.19$
Ivory Coast / gravity PL*	68.37	0.561	0.411	0.453	0.527	$\gamma = -0.51$
Ivory Coast / hierarchy PL*	51.18	0.315	0.376	0.313	0.676	$\gamma = -2.88$
Ivory Coast / hierarchy-admin PL*	80.80	0.684	0.449	0.555	0.415	$\gamma = -1.19$
Ivory Coast / gravity PL loc	54.03	0.267	0.374	0.240	0.752	$\gamma = -0.76$
Ivory Coast / hierarchy PL loc	48.69	0.276	0.371	0.275	0.716	$\gamma = -2.99$
Ivory Coast / hierarchy-admin PL loc	68.53	0.445	0.438	0.317	0.674	$\gamma = -1.99$

Table S7. Models ranked according to their average performance across all studied countries, for each benchmark measure (the deviance E being normalized by its value for the Hierarchy EXP loc model E_{ref}). Models are sorted according to $\langle rank_{E/E_{ref}} \rangle$ (note: this ranking does not take into account the number of parameters involved in the different models). The rows corresponding to models presented in the main text are highlighted.

Model	$\langle rank_{E/E_{ref}} \rangle$	$\langle rank_D \rangle$	$\langle rank_S \rangle$	$\langle rank_C \rangle$	$\langle rank_{corr} \rangle$	$\langle rank \rangle$
hierarchy EXP loc	1.80	3.00	2.20	3.20	3.80	2.80
gravity PL loc	2.20	1.80	2.00	2.20	3.00	2.24
hierarchy EXP	4.20	7.00	4.40	8.00	7.40	6.20
hierarchy EXP*	5.20	7.40	4.40	7.40	7.80	6.44
hierarchy PL loc	5.40	5.80	8.20	6.60	7.00	6.60
gravity PL	6.80	11.40	5.60	11.00	12.00	9.36
hierarchy-admin EXP loc	8.20	8.80	8.60	7.80	10.00	8.68
hierarchy PL	8.40	9.60	9.80	10.00	10.60	9.68
hierarchy-admin PL loc	8.80	8.40	9.80	8.00	10.00	9.00
hierarchy PL*	9.60	9.80	9.40	10.20	10.60	9.92
gravity PL*	10.40	13.40	7.80	12.00	12.80	11.28
gen. radiation	11.00	6.80	5.00	4.40	5.60	6.56
gravity EXP loc	11.60	13.60	12.20	13.20	13.60	12.84
hierarchy-admin EXP	13.60	14.60	12.40	14.00	14.40	13.80
hierarchy-admin EXP*	14.80	13.00	12.00	13.60	14.00	13.48
hierarchy-admin PL	15.20	14.60	14.60	15.80	16.20	15.28
hierarchy-admin PL*	16.40	13.20	13.80	15.40	15.80	14.92
gravity EXP	16.20	16.80	14.00	16.00	16.20	15.84
gravity EXP*	17.60	14.20	13.80	16.20	16.80	15.72
radiation	19.80	16.80	18.00	8.80	9.40	14.56

References

1. Rand, W. Objective criteria for the evaluation of clustering methods. *Journal of the American Statistical Association* **66**, pp. 846–850 (1971).
2. Jain, A. K. & Dubes, R. C. *Algorithms for clustering data* (Prentice-Hall, Englewood Cliffs, NJ., 1988).
3. Fowlkes, E. & Mallows, C. *A method for comparing two hierarchical algorithms*, vol. 78 (Journal of the American Statistical Association, 1983).
4. Ratti, C. *et al.* Redrawing the map of Great Britain from a network of human interactions. *PLoS ONE* **5**, e14248 (2010).
5. Sobolevsky, S. *et al.* Delineating geographical regions with networks of human interactions in an extensive set of countries. *PLoS ONE* **8**, e81707 (2013).
6. Sobolevsky, S., Campari, R., Belyi, A. & Ratti, C. General optimization technique for high quality community detection in complex networks. *Physical Review E* **90**, 012811 (2014).
7. Christaller, W. *Die zentralen Orte in Süddeutschland* (Wissenschaftliche Buchgesellschaft, 1933).
8. Christaller, W. *Central places in southern Germany: Translated from Die zentralen Orte in Süddeutschland* (Prentice Hall, 1966).
9. Lösch, A. *Die räumliche Ordnung der Wirtschaft* (G. Fischer, 1944).
10. Veneris, Y. *The informational revolution, cybernetics and urban modelling.* Ph.D. thesis, University of Newcastle upon Tyne (1984).
11. Simini, F., González, M., Maritan, A. & Barabási, A.-L. A universal model for mobility and migration patterns. *Nature* **484**, 96–100 (2012).
12. Simini, F., Maritan, A. & Néda, Z. Human mobility in a continuum approach. *PLoS ONE* **8**, e60069 (2013).
13. Flowerdew, R. & Aitkin, M. A method of fitting the gravity model based on the poisson distribution*. *Journal of Regional Science* **22**, 191–202 (1982).
14. Wilson, A. G. A statistical theory of spatial distribution models. *Transportation research* **1**, 253–269 (1967).
15. Sagarra, O., Vicente, C. P. & Dïaz-Guilera, A. Statistical mechanics of multiedge networks. *Physical Review E* **88**, 062806 (2013).
